# Dammarenediol II enhances etoposide‐induced apoptosis by targeting *O*‐GlcNAc transferase and Akt/GSK3β/mTOR signaling in liver cancer

**DOI:** 10.1002/1878-0261.70199

**Published:** 2025-12-30

**Authors:** Jaehoon Lee, Byung‐Cheol Han, Gi‐Bang Koo, Jihye Park, Mijin Kwon, Young Bin Park, Jae‐Mun Choi, Seung‐Ho Lee, Sangho Roh

**Affiliations:** ^1^ R&D Headquarter Korea Ginseng Corporation Gwacheon‐si Korea; ^2^ Cellular Reprogramming and Embryo Biotechnology Laboratory Dental Research Institute, Seoul National University School of Dentistry Seoul Korea; ^3^ Calici Co., Ltd., USA San Jose CA USA; ^4^ Calici Co., Ltd., Korea Daejeon Korea; ^5^ Department of Bio AI Convergence Chungnam National University Daejeon Korea; ^6^ Department of Food and Biotechnology Korea University Sejong Korea

**Keywords:** Akt signaling pathway, anticancer chemotherapy, apoptosis, dammarenediol II, etoposide, *O*‐GlcNAc transferase inhibitor

## Abstract

Combining chemotherapy with chemosensitizing agents is a common strategy to enhance anticancer efficacy while mitigating treatment‐related side effects. This study investigated the potential of dammarenediol II (DM2), a ginsenoside precursor, to enhance the anticancer effects of etoposide by downregulating *O*‐linked β‐N‐acetylglucosamine modification (*O*‐GlcNAcylation) and modulating the Akt signaling pathway in HepG2 human liver cancer cells. The effect of DM2 on *O*‐GlcNAcylation regulation was analyzed using Pharmaco‐Net, an artificial intelligence–driven drug screening platform and further validated using *O*‐GlcNAc transferase (OGT) activity assay. DM2 cotreatment enhanced etoposide's anticancer efficacy, which was quantitatively evaluated by viability, Annexin V binding, membrane integrity, and caspase‐3/7 activity assays in HepG2 cells. Results showed that DM2 reduced *O*‐GlcNAc levels by directly interacting with OGT, as confirmed through Pharmaco‐Net. Cotreatment with 40 μm DM2 and 20 μm etoposide produced synergistic anticancer effects, lowering etoposide's IC_50_ for cell viability by 2.29‐fold and its EC_50_ for caspase‐3/7 activity by 3.64‐fold. Mechanistically, DM2 dose‐dependently suppressed Akt/GSK3β/mTOR signaling. Using the Akt activator SC79, additional experiments confirmed that Akt signaling acts downstream of *O*‐GlcNAcylation regulated by etoposide and DM2. These effects were also observed in multiple human liver cancer cell lines, as well as in A549 lung and Caco‐2 colorectal cancer cells. This supports the broader anticancer and Akt‐inhibitory potential of DM2. This study is the first to demonstrate that DM2 enhances anticancer synergy by suppressing *O*‐GlcNAcylation and Akt signaling, highlighting its potential as a novel chemotherapy adjuvant.

## Graphical abstract

Etoposide induces DNA damage, activating p53‐dependent apoptosis via caspase‐3/7, which cleaves PARP1. Dammarenediol II enhances this apoptotic pathway by suppressing O‐GlcNAc transferase activity, further decreasing O‐GlcNAcylation. The reduction in O‐GlcNAc levels boosts p53‐driven apoptosis and influences the Akt/GSK3β/mTOR signaling pathway, facilitating cell death. Combined treatment with dammarenediol II and etoposide synergistically lowers O‐GlcNAcylation and strengthens apoptosis.

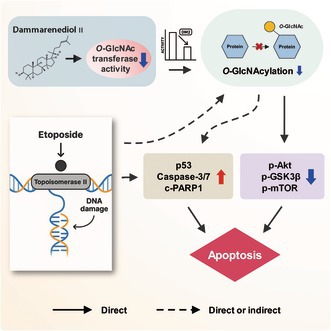

**Abbreviations**
AI: artificial intelligence
ATCC: American Type Culture Collection
BEP: bleomycin, etoposide, and cisplatin
DM2: dammarenediol II
FACS: fluorescence‐activated cell sorting
GPC3: glypican‐3
HBP: hexosamine biosynthetic pathway
HCC: hepatocellular carcinoma
MCE: MedChemExpress
OGA: *O*‐GlcNAcase
*O*‐GlcNAcylation: *O*‐linked β‐N‐acetylglucosamine modification
OGT: *O*‐GlcNAc transferase
PBS: phosphate‐buffered saline
SDs: standard deviations
Topo II: topoisomerase II
UDP: uridine diphosphate
VIP: etoposide, ifosfamide, and cisplatin

## 1 Introduction

*O*‐linked β‐N‐acetylglucosamine modification (*O*‐GlcNAcylation) is a reversible posttranslational modification, comparable to protein phosphorylation, that plays a key role in cell signaling. Although over 500 enzymes contribute to phosphorylation regulation, *O*‐GlcNAcylation is regulated by only two enzymes: *O*‐GlcNAc transferase (OGT) and *O*‐GlcNAcase (OGA) [1]. To date, >8000 human *O*‐GlcNAcylated proteins have been identified, predominantly in the nervous system, liver, skin, kidneys, intestines, and muscle tissue (in order of abundance, The *O*‐GlcNAc Database, v2.0) [2]. Dysregulation of *O*‐GlcNAcylation may contribute to diseases across various organs. Indeed, studies have linked *O*‐GlcNAcylation failure to physiological disruption, leading to the development or progression of skeletal muscle disorders [3], metabolic disorders [4], autoimmune diseases [5], and neurodegenerative diseases [6], underscoring the importance of understanding its mechanisms for therapeutic development. Targeting *O*‐GlcNAc‐regulating enzymes (e.g., OGT, OGA, and hexosaminidase) and associated pathways offers promising treatment strategies.

Liver cancer, the third leading cause of cancer deaths as of 2020, is projected to rise by 56.4% by 2040 [7], emphasizing the need for novel treatments. Global *O*‐GlcNAcylation levels are markedly elevated in human hepatocellular carcinoma (HCC) tissues compared with healthy liver tissues [8]. For example, *O*‐GlcNAcylation of serine 122 from the RACK1 protein promotes tumor growth, metastasis, and angiogenesis via protein stabilization [9], whereas *O*‐GlcNAcylation of hepatocyte growth factor–regulated tyrosine kinase substrate, a primary component of the endosomal sorting complex required for transport, enhances tumor progression *in vivo* and anticancer drug resistance in liver cancer cells [10]. The Akt signaling pathway, vital for cell survival, proliferation, and metabolism, is closely associated with cancer progression, including liver cancer, through its aberrant activation [11]. *O*‐GlcNAcylation has been shown to activate the PI3K/Akt/mTOR signaling pathway, which integrates mitogenic stimuli and nutrient status to promote cell cycle progression in the HepG2 human liver HCC cell line [12].

Etoposide, a chemotherapeutic agent that inhibits DNA topoisomerase II (Topo II), induces DNA damage and apoptosis in cancer cells. It is used as a standard treatment for cancers, such as small cell lung cancer, testicular cancer, and lymphoma. Owing to its limited efficacy and side effects, etoposide is often used in combination chemotherapies, minimizing chemotherapy resistance development while enhancing survival rates and treatment effects [13]. Typical combination therapies, for example, BEP (bleomycin, etoposide, and cisplatin) and VIP (etoposide, ifosfamide, and cisplatin), are particularly effective against high‐risk cancers, for example, testicular cancer [14]. However, these anticancer regimens have limitations. For example, BEP can cause pulmonary fibrosis, and VIP avoids pulmonary toxicity but may induce severe immunosuppression [15]. Therefore, developing flexible treatment options for various patient conditions remains critical.

Dammarenediol II (DM2) is an intermediate in producing dammarane‐type ginsenosides, bioactive compounds found in *Panax* species (e.g., ginseng). These ginsenosides exhibit diverse pharmacological effects, including anti‐inflammatory [16], anticancer [17], and neuroprotective properties [18]. Advances in metabolic engineering have enabled heterologous production of DM2 and its derivatives in microbial systems [19]. For instance, introducing *Panax ginseng* DM2 synthase into *Chlamydomonas reinhardtii* yielded up to 2.6 mg·L^−1^ DM2 through photosynthetic production under optimized conditions [20]. DM2 inhibits human carboxylesterases [21] and improves diabetic retinopathy in animal models [22]. Microbial production offers a sustainable and scalable platform as well as providing opportunities for structural modification to enhance DM2's pharmacological properties.

This study investigates DM2's potential to sensitize liver cancer cells to etoposide. The findings revealed that DM2 decreases OGT activity through direct molecular interaction, a conclusion validated using Pharmaco‐Net, an artificial intelligence (AI)‐driven, deep learning–based platform. DM2 reduced *O*‐GlcNAcylation and Akt activity in a dose‐dependent manner, enhancing etoposide's apoptosis‐inducing and cytotoxic effects in HepG2 cells. These effects extended to Hep3B, A549, and Caco‐2 cancer cells, confirming the Akt signaling pathway as a key mediator of the observed synergy. Overall, these results suggest that DM2 modulates *O*‐GlcNAcylation and Akt pathway activity, making it a promising adjuvant for improving chemotherapy efficacy.

## 2 Materials and methods

### 2.1 General methods and materials

The experiments followed methodologies established in prior studies [23]. DM2 was obtained from ChemScene (Monmouth Junction, NJ, USA) and prepared as a 20 mm stock solution in dimethyl sulfoxide (DMSO) for storage. Etoposide and Thiamet G were purchased from MedChemExpress (MCE; Monmouth Junction, NJ, USA) and prepared as 50 and 40 mm stock solutions in DMSO for storage, respectively. OSMI‐1 was obtained from GlpBio (Montclair, CA, USA) and prepared as a 40 mm stock solution in DMSO for storage. DM2, etoposide, OSMI‐1, and Thiamet G stock solutions were stored at −80 °C. Tween 20 and bisBenzimide H 33342 trihydrochloride (Hoechst 33342) were purchased from Sigma‐Aldrich (Saint Louis, MO, USA). Phalloidin‐iFluor™ 555 conjugate was acquired from Cayman Chemical (Ann Arbor, MI, USA). Antibodies were supplied from the following manufacturers: Akt (C67E7; monoclonal; #4691), p‐Akt (Ser473; D9E; monoclonal; #4060), GSK3β (27C10; monoclonal; #9315), p‐GSK3β (Ser9; monoclonal; #9336), mTOR (7C10; monoclonal; #2983), p‐mTOR (Ser2448; D9C2; monoclonal; #5536), *O*‐GlcNAc (monoclonal; #82332) for western blot analysis from Cell Signaling Technology (Danvers, MA, USA); *O*‐GlcNAc (RL2; monoclonal; #MA1‐072) for immunocytochemistry from Thermo Fisher Scientific (Waltham, MA, USA); OGT (ARC0790; monoclonal; #A3501), OGA (ARC65434; monoclonal; #A24124), cleaved‐PARP1 (ARC0091; monoclonal; #A19612) from ABclonal Technology (Woburn, MA, USA); and β‐actin (polyclonal; #ab8227) and p53 (PAb421; monoclonal; #NBP2‐62555) from Abcam (Cambridge, United Kingdom) and Novus Biologicals (Centennial, CO, USA), respectively.

### 2.2 Cell culture

HepG2 (RRID:CVCL_0027) and Hep3B (RRID:CVCL_0326) human liver hepatocellular carcinoma, Caco‐2 (RRID:CVCL_0025) human colorectal adenocarcinoma, and A549 (RRID:CVCL_0023) human lung carcinoma cell lines were obtained from the American Type Culture Collection (ATCC, Manassas, VA, USA). Huh7 (RRID:CVCL_0336), SK‐HEP1 (RRID:CVCL_0525), SNU‐182 (RRID:CVCL_0090), and SNU‐387 (RRID:CVCL_0250) human liver carcinoma cell lines were obtained from the Korean Cell Line Bank (KCLB, Seoul, South Korea). All cell lines used in this study were authenticated within the past two years. Human cell lines were authenticated by the cell line providers using short tandem repeat profiling, and authentication reports were provided by the suppliers. No additional genetic manipulation or prolonged passaging was performed after authentication. Cell lines were used at low passage numbers to minimize the risk of genetic drift or cross‐contamination. All experiments were performed with mycoplasma‐free cells, and all cell lines were confirmed to be mycoplasma‐negative prior to use using the MycoStrip™ Mycoplasma Detection Kit (InvivoGen, San Diego, CA, USA), according to the manufacturer's instructions. HepG2, Huh7, Hep3B, and Caco‐2 cells were cultured in Eagle's minimum essential medium (ATCC) containing 1.0 g·L^−1^ d‐glucose, 1.5 g·L^−1^ sodium bicarbonate, 110.0 mg·L^−1^ sodium pyruvate, 292.0 mg·L^−1^ L‐glutamine, 10% fetal bovine serum (FBS), and 1% penicillin/streptomycin (P/S). Huh7, SNU‐182, and SNU‐387 were cultured in RPMI 1640 Medium (Thermo Fisher Scientific) containing 2.0 g·L^−1^ D‐glucose, 2.0 g·L^−1^ sodium bicarbonate, 300.0 mg·L^−1^ L‐glutamine, 10% FBS, and 1% P/S. A549 cells were cultured in Kaighn's modification of Ham's F‐12 Medium (ATCC) containing 1.26 g·L^−1^ D‐glucose, 1.5 g·L^−1^ sodium bicarbonate, 220.0 mg·L^−1^ sodium pyruvate, 292.2 mg·L^−1^ L‐glutamine, 10% FBS, and 1% P/S. All cells were cultured at 37 °C in a humidified incubator with a 5% CO_2_ atmosphere.

### 2.3 Cell viability assay

HepG2, Huh7, SK‐HEP1, SNU‐182, SNU‐387, Hep3B, A549, and Caco‐2 cells were seeded in 96‐well plates at 1.0 × 10^4^ cells per well and incubated for 24 h. Subsequently, the medium was replaced with varying concentrations of the test drugs. Cell viability was evaluated after an additional 24 h using the D‐Plus™ CCK Cell Viability Assay Kit (Dongin LS, Seoul, South Korea).

### 2.4 Annexin V binding and membrane integrity assay

The RealTime‐Glo™ Annexin V Apoptosis and Necrosis Assay Kit (Promega, Madison, WI, USA) was used to evaluate drug effects on apoptosis and necrosis. HepG2, Huh7, SK‐HEP1, SNU‐182, and SNU‐387 cells were seeded at 1.0 × 10^4^ cells per well in solid white 96‐well plates and incubated for 24 h. The medium was then replaced with fresh medium containing the drugs and assay kit components according to the manufacturer's instructions. Luminescence and fluorescence signals were measured using the GloMax^®^ Discover Microplate Reader (Promega).

### 2.5 Viability (GF‐AFC), cytotoxicity (bis‐AAF‐R110), and caspase‐3/7 activity assays

Drug effects on cell viability, cytotoxicity, and caspase‐3/7 activity in HepG2 cells were evaluated using the ApoTox‐Glo™ Triplex Assay Kit (Promega). HepG2 cells were seeded at 1.0 × 10^4^ cells per well in solid white 96‐well plates and incubated for 24 h. Then, the medium was replaced with fresh medium containing varying drug concentrations, and the cells were incubated for an additional 24 h. GF‐AFC and bis‐AAF‐R110 substrates were then added in accordance with the manufacturer's instructions, and fluorescence was measured after 2 h (viability: 400/505 nm excitation/emission; cytotoxicity: 485/520 nm excitation/emission). Subsequently, a luminogenic caspase‐3/7 substrate was added, and the plates were incubated for another 2 h at 37 °C. Luminescence and fluorescence signals were recorded using the GloMax^®^ Discover Microplate Reader (Promega).

### 2.6 Western blot and quantitative analyses

Proteins were extracted using Cell Culture Lysis 1× Reagent (Promega) containing protease and phosphatase inhibitors cocktails (MCE). Protein signals were developed using electrochemiluminescence western blot analysis substrates (Thermo Fisher Scientific). HepG2, Huh7, SK‐HEP1, SNU‐182, SNU‐387, Hep3B, A549, and Caco‐2 cells were collected and lysed on ice for 5 min using Cell Culture Lysis 1X Reagent (Promega) supplemented with protease and phosphatase inhibitors (MCE). Insoluble debris was removed by centrifugation at 15 000 ***g*** for 20 min 4 °C, and the protein concentration in the resulting supernatants was measured using the Pierce™ BCA Protein Assay Kit (Thermo Fisher Scientific). Proteins (10–20 μg) were separated on the Bolt™ Bis‐Tris Plus Gel system (Thermo Fisher Scientific) and transferred onto polyvinylidene difluoride membranes (Bio‐Rad, Hercules, CA, USA). The membranes were blocked in 0.05% Tween 20 Tris‐buffered saline (TBST) containing 3%–5% bovine serum albumin (BSA) for 1 h at room temperature and then incubated overnight at 4 °C with diluted primary antibodies in 5% BSA–TBST. After three washes with TBST, membranes were incubated with horseradish peroxidase‐conjugated secondary antibodies in 5% BSA–TBST for 1 h at room temperature. Protein signals were visualized using ECL substrates (Thermo Fisher Scientific) and analyzed using Fusion FX6.0 (Vilber, Collégien, France). ImageJ software (version 1.54 g) was used to quantify the band intensity from western blot analysis.

### 2.7 Immunocytochemistry

HepG2 cells were incubated on gelatin‐coated coverslips (SPL Life Sciences, Pocheon‐si, South Korea), treated with the indicated drugs, and incubated on the coverslips with primary antibodies and secondary antibodies in 10% normal donkey serum. Stained cells were visualized using LSM 900 with Airyscan 2 confocal microscopes (Carl Zeiss, Oberkochen, Germany). HepG2 cells were seeded at an appropriate density on gelatin‐coated coverslips (SPL Life Sciences) and treated with the indicated drugs. Cells were fixed with 4% paraformaldehyde for 20 min, permeabilized with 0.2% Triton X‐100 for 5 min, blocked with 10% normal donkey serum (Abcam) for 1 h. The coverslips were then incubated overnight with primary antibodies diluted in 10% normal donkey serum. After three washes with phosphate‐buffered saline (PBS) containing 0.1% Tween 20 (PBST), cells were incubated with Alexa Fluor 488–conjugated secondary antibodies for 30 min. Following additional washes with PBST, the coverslips were incubated with phalloidin‐iFluor™ 555 conjugate and Hoechst 33342 for 10 min. Finally, the coverslips were mounted on slides using ProLong™ Glass Antifade Mountant (Thermo Fisher Scientific), and the stained cells were visualized using LSM 900 with Airyscan 2 confocal microscopes (Carl Zeiss).

### 2.8 Real‐time quantitative polymerase chain reaction (RT‐qPCR)

HepG2 cells were seeded at a density of 1.0 × 10^6^ cells per well in 6‐well plates and incubated for 24 h. The culture medium was then replaced with fresh medium containing the indicated drug concentrations, followed by an additional 24‐h incubation. Total RNA was isolated using the QIAwave RNA Mini Kit (Qiagen, Hilden, Germany) according to the manufacturer's instructions. cDNA was synthesized from 1 μg of total RNA using the iScript™ Reverse Transcription Supermix (Bio‐Rad) on a T100 Thermal Cycler (Bio‐Rad). The reverse transcription conditions were as follows: priming at 25 °C for 5 min, reverse transcription at 46 °C for 20 min, and inactivation at 95 °C for 1 min. RT‐qPCR was performed using AccuPower^®^ 2X GreenStar™ qPCR MasterMix (Bioneer, Daejeon, South Korea) on a CFX96 Touch Real‐Time PCR Detection System (Bio‐Rad). Primers were purchased from Bioneer, and detailed primer sequences are provided in Table S1. Relative mRNA expression levels were calculated using the ΔΔCt method and normalized to GAPDH.

### 2.9 Protein structure–based drug screening using Pharmaco‐net

The crystal structure of human OGT was obtained from the Protein Data Bank (PDB ID: 4GYW). Protein structure–based drug screening was performed using the various modules of Pharmaco‐Net, an AI‐SaaS platform (https://pharmaco-net.org/) powered by Calici Co. The X‐ray crystal structure of the human *O*‐GlcNAc Transferase was secured from the Protein Data Bank (PDB ID: 4GYW). Protein structure–based drug screening was conducted using various modules on the Pharmaco‐Net, an AI‐SaaS platform (https://pharmaco-net.org/) powered by Calici Co. The active site within the human *O*‐GlcNAc Transferase was identified using the PocketFinder module. Thereafter, *in silico* docking for calculating a binding energy value between the human *O*‐GlcNAc Transferase and natural compound libraries (commercialized or in‐house libraries) was performed using the AI‐Dock module. The actual binding affinities (μm) were predicted using the 4DCNN model in the DeepCalici‐Plus module. The 3D structure data of the protein–chemical complex image was visualized using the Interaction Viewer module or PyMol v3.0.3 software.

### 2.10 OGT activity assay

OGT activity was measured using the UDP‐Glo™ Glycosyltransferase Assay Kit (Promega) following the manufacturer's instructions. Uridine diphosphate (UDP)‐GlcNAc (Promega) served as the UDP–sugar substrate, and the OGT peptide substrate (KKKYPGGSTPVSSANMM) was obtained from AnaSpec (Fremont, CA, USA). Recombinant human OGT protein was purchased from R&D Systems (Minneapolis, MN, USA). Reactions were conducted in 1× OGT reaction buffer containing 25 mm Tris (pH 7.5), 12.5 mm MgCl_2_, 0.06 mg·mL^−1^ BSA, and 1 mm dithiothreitol.

### 2.11 Fluorescence‐activated cell sorting (FACS)

HepG2 cells were stained using the Annexin V Apoptosis Detection Kit with 7‐AAD (STEMCELL Technologies, Vancouver, BC, Canada) following the manufacturer's instructions and analyzed using the FACSLyric™ System (BD Biosciences, Franklin Lakes, NJ, USA). HepG2 cells were seeded at 1.0 × 10^6^ cells per well in 6‐well plates in the medium described above. After 24 h, the medium was replaced with fresh medium containing the specified concentrations of etoposide and DM2, and the cells were incubated for an additional 24 h. Subsequently, cells were then collected and stained using the Annexin V Apoptosis Detection Kit with 7‐AAD (STEMCELL Technologies, Vancouver, BC, Canada), according to the manufacturer's protocol, and analyzed using the FACSLyric™ System (BD Biosciences, Franklin Lakes, NJ, USA). The gating for Annexin V and 7‐AAD was performed using HepG2 cells treated with 100 μm etoposide for 24 h.

### 2.12 TUNEL assay

HepG2 cells cultured in 6‐well plates were fixed with 4% formaldehyde for 30 min, permeabilized using PBS containing 0.2% Triton X‐100 and 0.5% BSA, and incubated with TUNEL reaction mixture (Abbkine, Atlanta, GA, USA) for 2 h at 37 °C. The cells were then stained with phalloidin‐iFluor™ 555 conjugate and 4′,6‐diamidino‐2‐phenylindole for 10 min at room temperature. After PBS washes, the cells were visualized using LSM 900 with Airyscan 2 confocal microscopes.

### 2.13 Quantitative detection of Akt phosphorylation

Lumit^®^ Immunoassay Cellular Systems (Promega) was used to evaluate normalized p‐Akt (Ser473) in HepG2 cells. The detected p‐Akt levels were normalized to viable cell number determined using the GF‐AFC substrate. Additional details are provided in Doc. S1.

### 2.14 Statistical analysis

Statistical analyses were conducted using SPSS Statistics version 20.0 (IBM Corp., Armonk, NY, USA) and GraphPad Prism 10 (GraphPad Software Inc., La Jolla, CA, USA). Results are expressed as means ± standard deviations (SDs). Data were analyzed using analysis of variance followed by Tukey's honest significant difference *post hoc* test, with significance set at *P* < 0.05.

## 3 Results

### 3.1 DM2 dose‐dependently decreases *O*‐GlcNAc levels in HepG2 cells

**Fig. 1:**
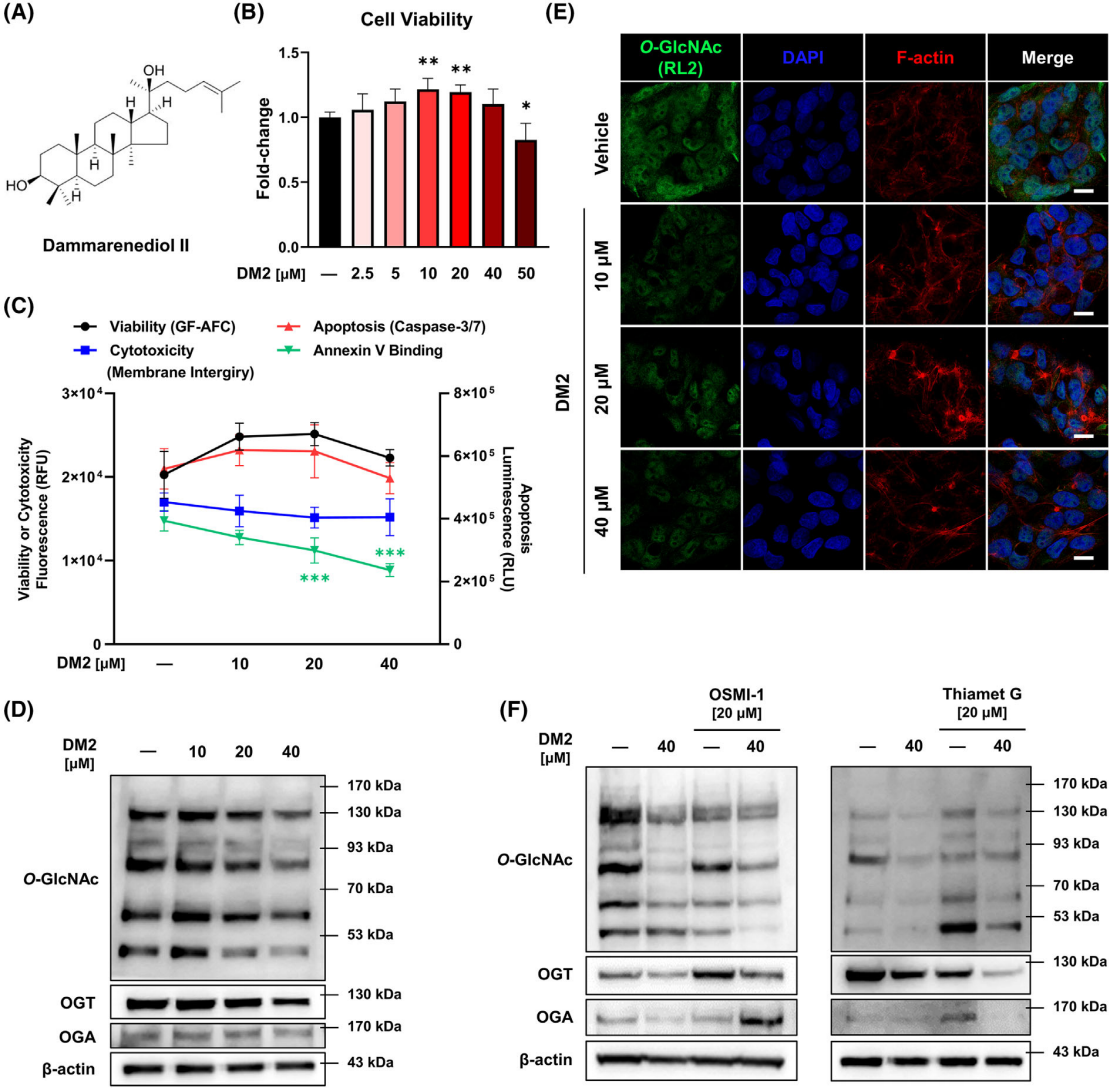
Dammarenediol II (DM2) reduces *O*‐GlcNAc levels in HepG2 cells in a concentration‐dependent manner. (A) Molecular structure of DM2. (B) DM2's impact on HepG2 cell viability based on a CCK cell viability assay (*n* = 6) after 24 h of treatment: cell viability remains unaffected at concentrations up to 40 μm but is reduced at 50 μm. (C) Multiple analyses, including GF‐AFC cell viability (*n* = 5), membrane integrity (*n* = 5), caspase‐3/7 activity (*n* = 4), and Annexin V binding (*n* = 5) assays, showing no significant effects of DM2 on cell viability or cytotoxicity. (D) Western blot analysis indicating that DM2 treatment for 24 h reduces *O*‐GlcNAc levels in HepG2 cells (*n* = 3). (E) Immunocytochemistry analysis using the *O*‐GlcNAc–specific antibody RL2 (630×; scale bar: 10 μm), corroborating the findings in (D). (F) Results of DM2 cotreatment with OSMI‐1, an *O*‐GlcNAc transferase (OGT) inhibitor, or Thiamet G, an *O*‐GlcNAcase (OGA) inhibitor, for 24 h: DM2 enhances OSMI‐1–induced reductions in *O*‐GlcNAc levels and counteracts Thiamet G–induced increases (*n* = 3). *P < 0.05, **P < 0.01, and ***P < 0.001 versus the control condition. Data represent means ± SD from at least three independent experiments. Statistical significance was determined with one‐way ANOVA with *post hoc* Tukey test.

DM2 (molecular formula: C_30_H_52_O_2_) has a molecular weight of 444.73 (Fig. 1A and Table 1). To evaluate its effect on HepG2 cells, cell viability was measured after 24 h of treatment. Exposure to concentrations of DM2 up to 40 μm resulted in no significant reduction in cell viability, but a significant decline was observed at 50 μm (Fig. 1B). Similarly, 24 h of DM2 treatment did not significantly affect cell viability or cytotoxicity (Fig. 1C). Analysis of *O*‐GlcNAc levels in HepG2 cells showed that DM2 decreased *O*‐GlcNAc levels in a concentration‐dependent manner (Fig. 1D,E, Fig. S1). Furthermore, 40 μm DM2 reduced OGT expression. Notably, OGT mRNA expression is significantly increased following 24 h treatment with 40 μm DM2 compared to the untreated control, whereas OGA expression shows no significant change (Fig. S2). To clarify DM2's regulatory role in *O*‐GlcNAcylation, cotreatment with OSMI‐1, an OGT inhibitor, or Thiamet G, an OGA inhibitor, was performed for 24 h. DM2 enhanced the *O*‐GlcNAc downregulation induced by OSMI‐1 and reversed the *O*‐GlcNAc upregulation induced by Thiamet G (Fig. 1F, Fig. S3). Quantitative analysis of western blot data showed that both DM2 and OSMI‐1 treatments for 24 h reduced O‐GlcNAc levels; however, DM2 decreased the expression of OGT, whereas OSMI‐1 increased OGT expression. Interestingly, OSMI‐1 and Thiamet‐G treatment respectively increased or decreased OGT protein expression, but there were no changes observed at the mRNA level. However, the cotreatment with DM2 significantly increased OGT expression. In contrast, DM2, OSMI‐1, or Thiamet‐G alone did not induce any changes in OGA expression, and only the cotreatment of DM2 and Thiamet‐G significantly increased OGA expression (Fig. S4). These findings suggest that DM2 reduces *O*‐GlcNAc levels through physiological regulation.

**Table 1:** Molecular properties of dammarenediol II.

	CAS number	Molecular formula	Molecular weight	SMILES
Dammarenediol II	14 351–29‐2	C_30_H_52_O_2_	444.73	CC(=CCC[C@@](C)([C@H]1CC[C@@]2([C@@H]1CC[C@H]3[C@]2(CC[C@@H]4[C@@]3(CC[C@@H](C4(C)C)O)C)C)C)O)C

### 3.2 DM2 suppresses *O*‐GlcNAc levels through direct interaction with OGT

**Fig. 2:**
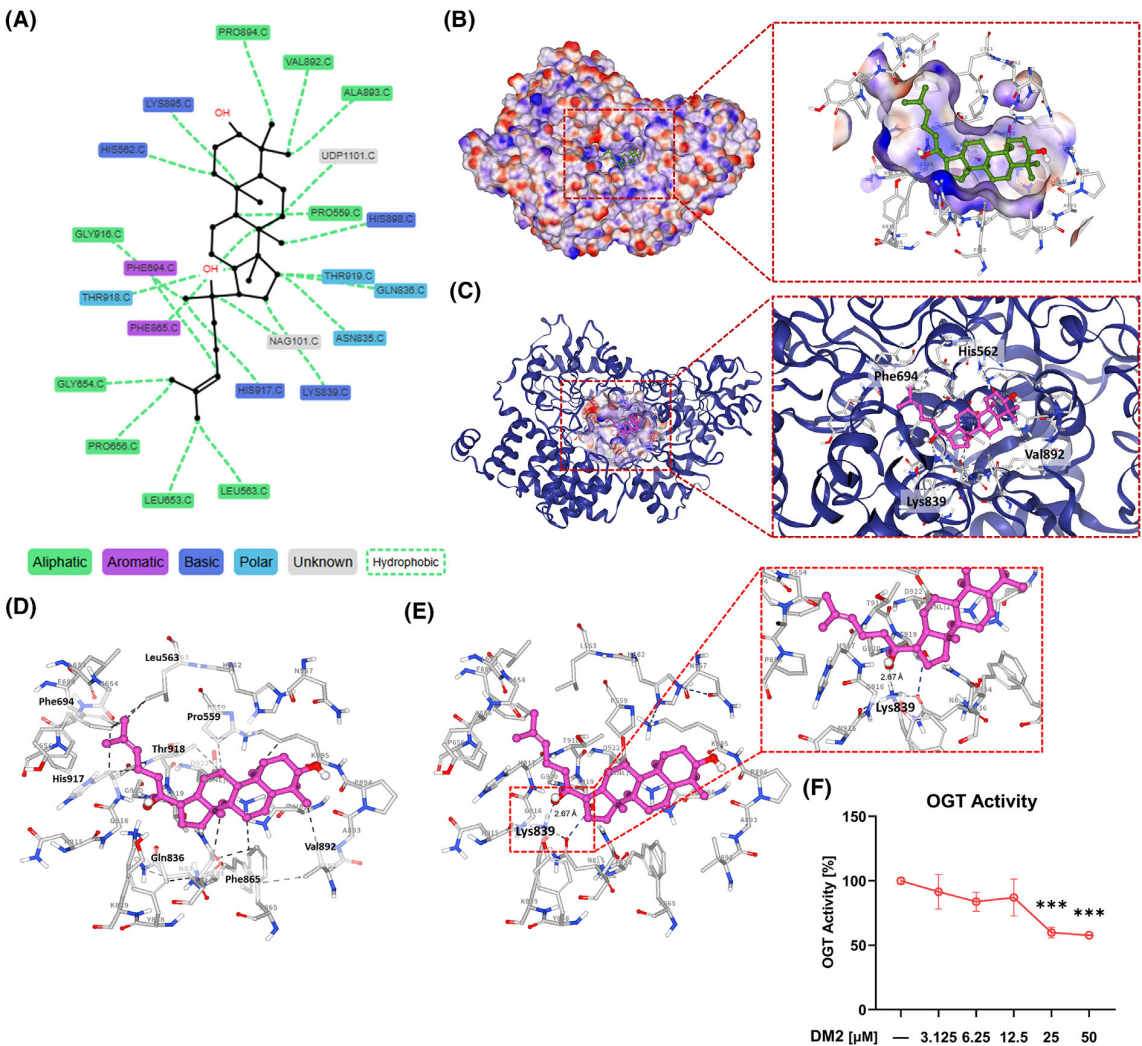
DM2 inhibits *O*‐GlcNAc levels by directly interacting with OGT. The Pharmaco‐Net platform was used for the following *in silico* analyses. (A) A 2D interaction map showing the specific amino acid residues in the OGT protein interacting with DM2 (*n* = 3). (B, C) 3D electrostatic potential and cartoon models confirming DM2 binding to OGT's active site. (D, E) Hydrophobic interactions and hydrogen bonding between DM2 and OGT according to *in silico* analysis. (F) Results of the OGT activity assay using OGT peptide substrate and UDP‐GlcNAc: 25 μm DM2 reduces OGT activity by ~40.17% (*n* = 4). The red dashed boxes represent a magnified view of the molecular interactions between DM2 and the active site of OGT. ***P < 0.001 versus the control condition. Data represent means ± SD from at least three independent experiments. Statistical significance was determined performed with one‐way ANOVA with *post hoc* Tukey test.

To determine whether DM2's effect on *O*‐GlcNAc downregulation involves direct interaction with OGT, Pharmaco‐Net was employed for *in silico* analysis. The X‐ray crystal structure of human OGT in complex with UDP and a glycopeptide (PDB ID: 4GYW) was retrieved from the Protein Data Bank. A 2D interaction map generated through Pharmaco‐Net illustrated DM2's interaction with specific OGT amino acid residues (Fig. 2A). Additionally, 3D electrostatic potential and cartoon models confirmed DM2 binding at OGT's active site (Fig. 2B,C). Furthermore, *in silico* analysis identified hydrophobic interactions and hydrogen bonding between DM2 and OGT (Fig. 2D,E). The Pharmaco‐Net platform predicted a binding energy of −5.83 kcal·mol^−1^ using the AI‐Dock module and a binding affinity of 0.0861 μm through the DeepCalici‐Plus module (Table 2). Consistently, activity assays involving OGT peptide substrate and UDP‐GlcNAc demonstrated that 25 μm DM2 reduced OGT activity by approximately 40.17% (Fig. 2F).

**Table 2:** Binding properties with *O*‐GlcNAc transferase.

	Binding energy (AI‐dock module)	Binding affinity value (DeepCalici‐plus module)
Dammarenediol II	−5.83 kcal·mol^−1^	0.0861 μm
OSMI‐1	−6.36 kcal·mol^−1^	0.0203 μm

### 3.3 DM2 enhances the synergistic anticancer effects of etoposide by reducing *O*‐GlcNAc levels across multiple cancer cell lines

**Fig. 3:**
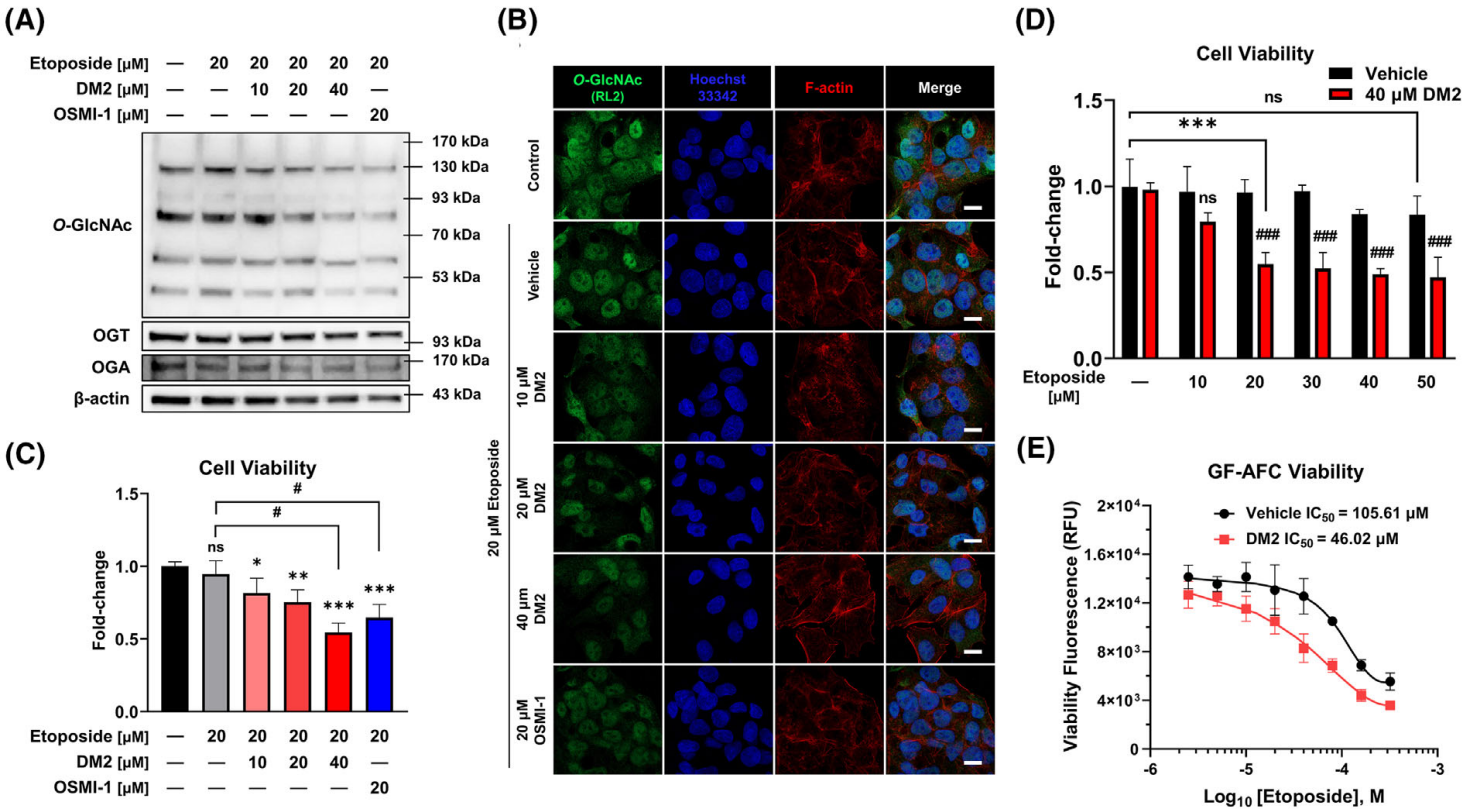
DM2 amplifies the synergistic anticancer effects of etoposide by downregulating *O*‐GlcNAc levels. (A, B) Analysis of *O*‐GlcNAc levels in HepG2 cells: cotreatment with etoposide and DM2 reduces *O*‐GlcNAc levels (630×; scale bar: 10 μm). (C) Cell viability assay results indicating the synergistic anticancer effects of DM2 on HepG2 cells (*n* = 5): etoposide–DM2 cotreatment significantly enhances this effect in a dose‐dependent manner compared to 20 μm etoposide alone. (D) Cell viability assay results showing that DM2 cotreatment at a fixed concentration of 40 μm significantly increases HepG2 sensitivity to etoposide (*n* = 3). (E) GF‐AFC assay results indicating a 2.29‐fold reduction in the IC_50_ value of etoposide (from 105.61 to 46.02 μm) with 40 μm DM2 (*n* = 4). *P < 0.05, **P < 0.01, and ***P < 0.001 versus the control condition; (C) ^#^ *P* < 0.05 versus the etoposide‐only treatment group; (D) ^###^ *P* < 0.001 versus the vehicle control in the etoposide treatment group at the same concentration. Data represent means ± SD from at least three independent experiments. Statistical significance was determined with one‐way or two‐way ANOVA with *post hoc* Tukey test. ns, not significant.

*O*‐GlcNAc levels analysis following DM2 and etoposide cotreatment in HepG2 cells confirmed a reduction in *O*‐GlcNAc levels (Fig. 3A,B). We previously found that downregulation of *O*‐GlcNAcylation through OSMI‐1–induced OGT inhibition increases the sensitivity of HepG2 cells to etoposide [23]. To understand the regulatory mechanism, we co‐treated etoposide with DM2 or OSMI‐1 and analyzed OGT and OGA mRNA expression by RT‐qPCR. Etoposide significantly increased the expression of OGT and OGA, and this increase was further enhanced by cotreatment with DM2. However, OSMI‐1 did not induce any additional differences (Fig. S5). To determine whether DM2 exerts similar synergistic anticancer effects, cell viability assays were performed. Results showed that 40 μm DM2 cotreatment significantly enhanced etoposide's anticancer effects in a dose‐dependent manner, compared to treatment with 20 μm etoposide alone (Fig. 3C). When 40 μm of DM2 was combined with etoposide, the anticancer effect increased significantly (Fig. 3D), although minimal additional effect was observed at etoposide concentrations exceeding 20 μm. The GF‐AFC viability assay further demonstrated that in the presence of 40 μm DM2, etoposide's IC_50_ value for HepG2 cell viability decreased by approximately 2.29‐fold to 46.02 μm (Fig. 3E).

### 3.4 DM2 augments etoposide‐induced apoptosis through synergistic activation of apoptotic pathways in liver cancer cells

**Fig. 4:**
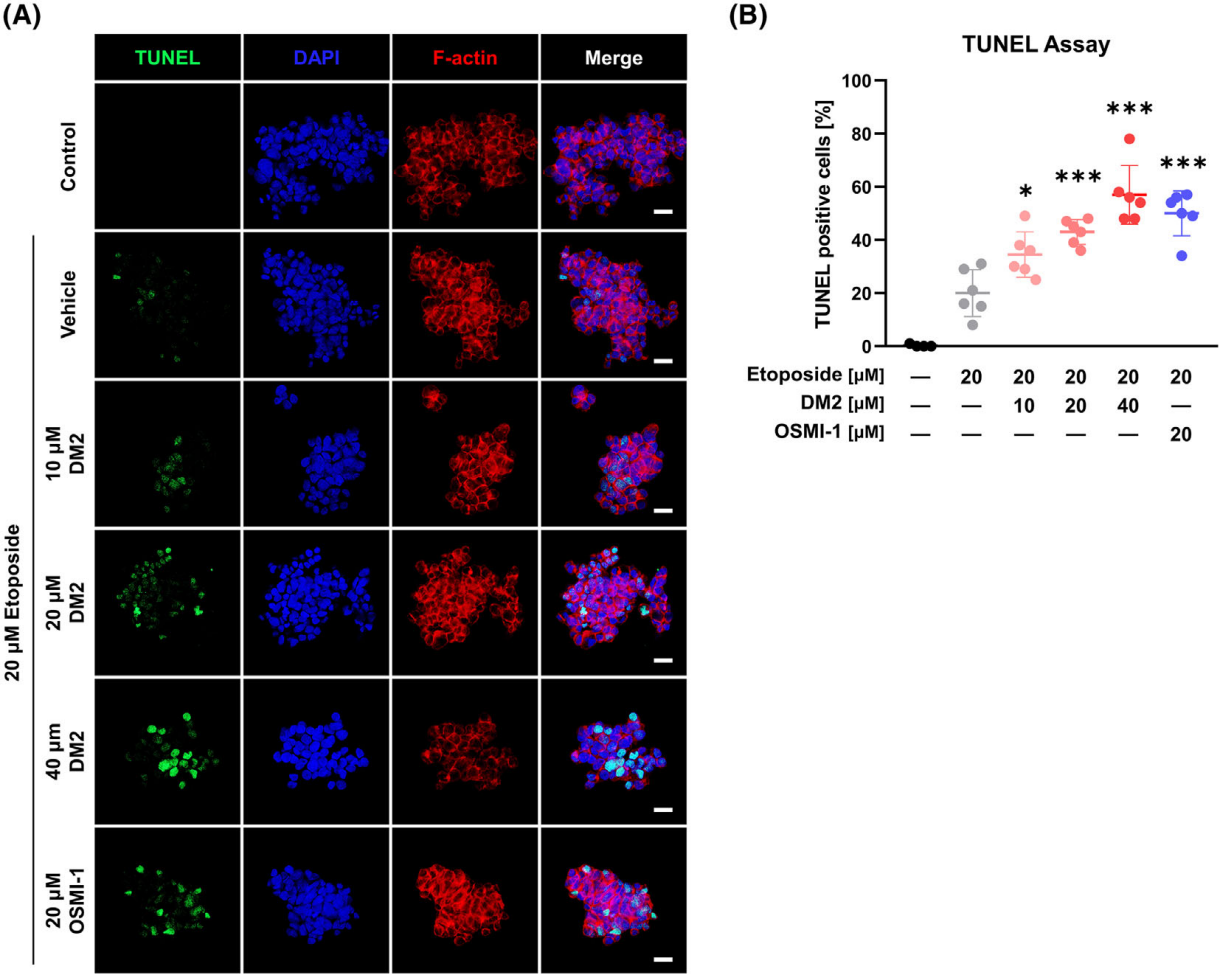
Cotreatment with etoposide and DM2 induces DNA fragmentation, contributing to apoptosis. (A, B) TUNEL assay results revealing dose‐dependent DNA fragmentation in HepG2 cells following 24 h of treatment with etoposide and DM2: DM2 enhances etoposide‐induced apoptosis (*n* = 6; 200×; white scale bar: 20 μm). *P < 0.05 and ***P < 0.001 versus the etoposide‐only treatment (mean ± SD). Statistical significance was determined with one‐way ANOVA with *post hoc* Tukey test.

**Fig. 5:**
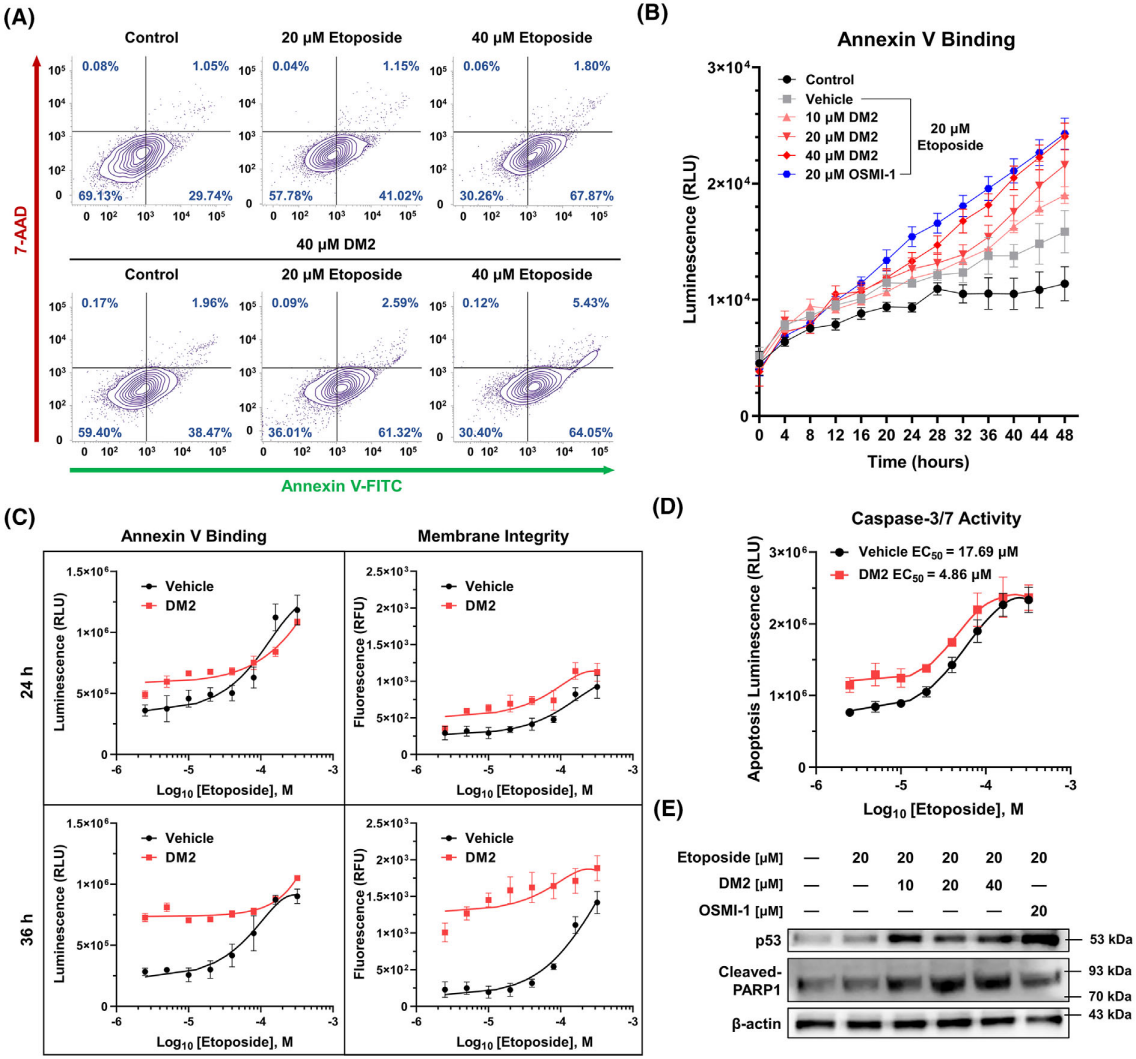
Cotreatment with etoposide and DM2 enhances apoptosis and reduces the concentration required for cytotoxicity. (A) FACS analysis with Annexin V and 7‐AAD staining: cotreatment with etoposide and DM2 for 24 h significantly enhances apoptosis compared with etoposide treatment alone. (B) Real‐time analysis with Annexin V: cotreatment with DM2 amplifies the apoptosis‐inducing effect of etoposide over time (*n* = 4). (C; top panel) A 24 h cotreatment with 40 μm DM2 effectively lowers the etoposide concentration required to induce apoptosis and cytotoxicity in HepG2 cells (*n* = 4). (C; bottom panel) After 36 h of treatment, apoptosis and cytotoxicity reach saturation at substantially lower etoposide concentrations during cotreatment with DM2 in HepG2 cells (*n* = 4). (D) Caspase‐3/7 activity analysis showing a 3.64‐fold reduction in the EC_50_ value of etoposide (from 17.69 to 4.86 μm) with 40 μm DM2 (*n* = 4). (E) Western blot analysis showing elevated expression of p53 and cleaved‐PARP1 following 24 h of cotreatment with 20 μm etoposide and 40 μm DM2 compared to etoposide treatment alone (*n* = 3). Data represent means ± SD from at least three independent experiments.

To determine whether the synergistic anticancer effects of DM2 occurred via enhancement of etoposide's apoptosis‐inducing ability, a TUNEL assay was performed. Cotreatment with DM2 significantly increased the percentage of TUNEL‐positive cells in a dose‐dependent manner (Fig. 4A,B). FACS analysis, performed for validation, indicated that etoposide‐induced phosphatidylserine translocation was more prominent with the 40 μm DM2 cotreatment (Fig. 5A). Furthermore, cotreatment with DM2 increased late‐phase apoptosis within 24 h of treatment, as confirmed via real‐time Annexin V analysis, which highlighted the cotreatment's progressively greater apoptotic effect over time (Fig. 5B). Dose–response curves revealed that 40 μm DM2 markedly reduced the etoposide concentration required to induce apoptosis and cytotoxicity in HepG2 cells after 24 h (Fig. 5C; top panel). By 36 h, apoptosis and cytotoxicity plateaued at much lower etoposide concentrations when DM2 was present (Fig. 5C; bottom panel). Mechanistic studies showed that DM2 decreased the EC_50_ value of etoposide on caspase‐3/7 activity from 17.69 to 4.86 μm, around a 3.64‐fold reduction (Fig. 5D). Correspondingly, cotreatment with DM2 and etoposide increased the expression of cleaved‐PARP1 and p53, markers of apoptosis activation (Fig. 5E).

**Fig. 6:**
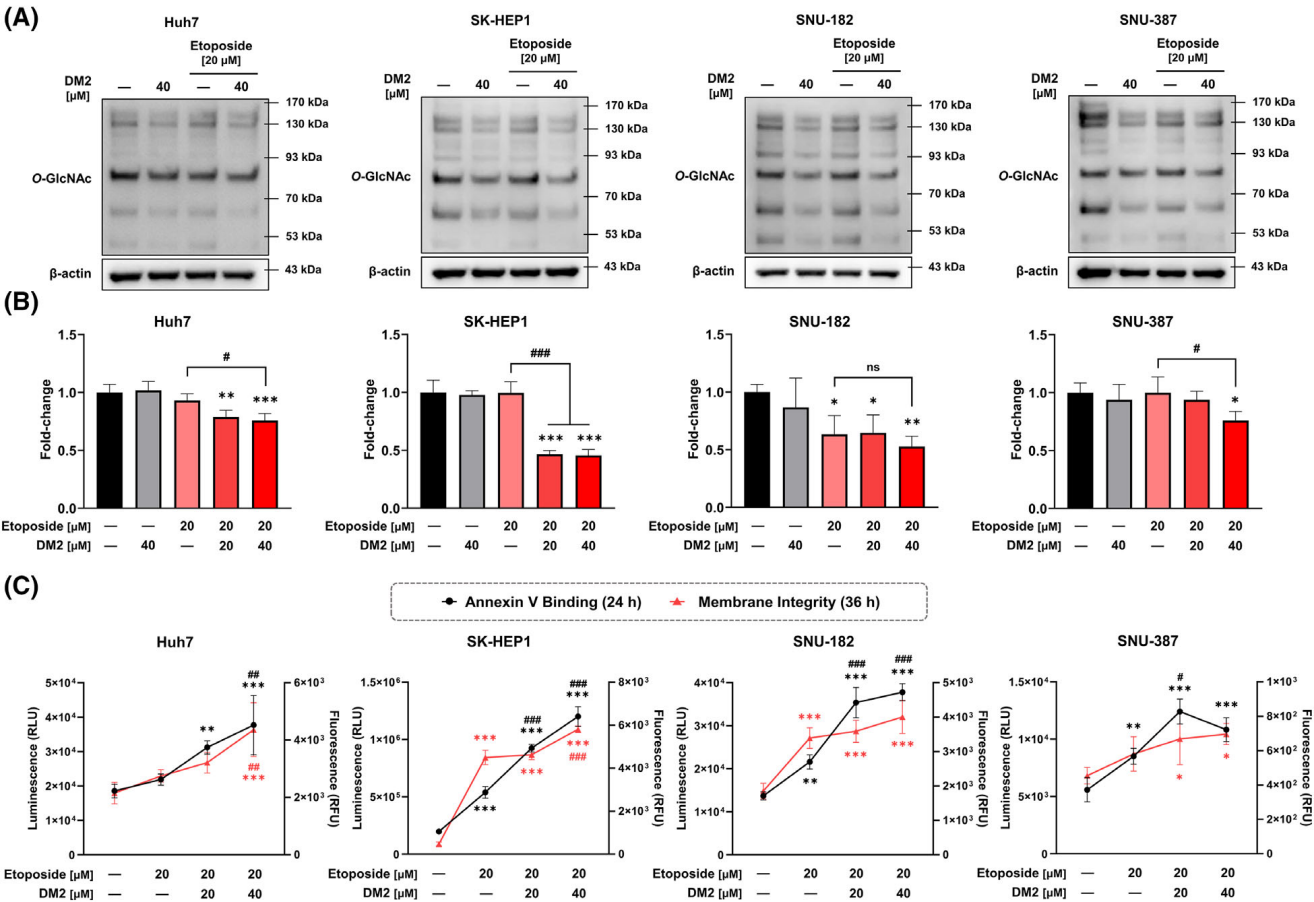
Cotreatment with etoposide and DM2 induces apoptosis and modulates cytotoxicity across multiple human liver cancer cell lines. (A) Western blot analysis of *O*‐GlcNAc levels after 24 h of DM2 treatment in Huh7, SK‐HEP1, SNU‐182, and SNU‐387 cells: DM2 reduces *O*‐GlcNAc levels in all tested liver cancer cell lines (*n* = 3). (B) Cell viability assay showing that DM2 synergistically enhances etoposide‐induced cytotoxicity in Huh7, SK‐HEP1, and SNU‐387 cells, but not in SNU‐182 cells (*n* = 4). (C) Annexin V analysis indicating that DM2 cotreatment for 24 h significantly increases apoptosis in all tested cell lines (*n* = 4). (D) After 36 h of treatment, increased cytotoxicity is observed only in Huh7 and SK‐HEP1 cells, suggesting cell line‐specific differences in temporal sensitivity to combination therapy (*n* = 4). *P < 0.05, **P < 0.01, and ***P < 0.001 versus the control condition; ^#^ *P* < 0.05, ^##^ *P* < 0.01, and ^###^ *P* < 0.001 versus the etoposide‐only treatment group. Data represent means ± SD from at least three independent experiments. Statistical significance was determined with one‐way ANOVA with *post hoc* Tukey test. ns, not significant.

To determine whether the synergistic anticancer effects of DM2 observed in HepG2 cells could be extended to other human liver cancer cell lines, additional experiments were conducted using Huh7, SK‐HEP1, SNU‐182, and SNU‐387 cells. Similar to the results in HepG2 cells, analysis of *O*‐GlcNAc levels revealed that DM2 treatment reduced *O*‐GlcNAc levels across all tested cell lines (Fig. 6A). Cell viability assay results demonstrated that, except for the SNU‐182 cells, DM2 synergistically enhanced the anticancer activity of etoposide after 24 h of cotreatment (Fig. 6B). While cotreatment with DM2 for 24 h significantly increased apoptotic cell death in all tested cell lines (Fig. 6C), extended treatment up to 36 h led to a marked increase in cytotoxicity only in Huh7 and SK‐HEP1 cells, suggesting cell line‐specific differences in the time‐dependent response to the combination therapy. Furthermore, DM2 treatment in Hep3B, A549, and Caco‐2 cells confirmed its broad anticancer potential through modulation of *O*‐GlcNAcylation and enhancement of etoposide‐induced cytotoxicity. While the cytotoxic effect in Caco‐2 cells did not reach statistical significance, the same decreasing tendency was consistently observed, suggesting a similar biological response (Fig. S6A,B).

### 3.5 DM2 inhibits the Akt signaling pathway in a dose‐dependent manner

**Fig. 7:**
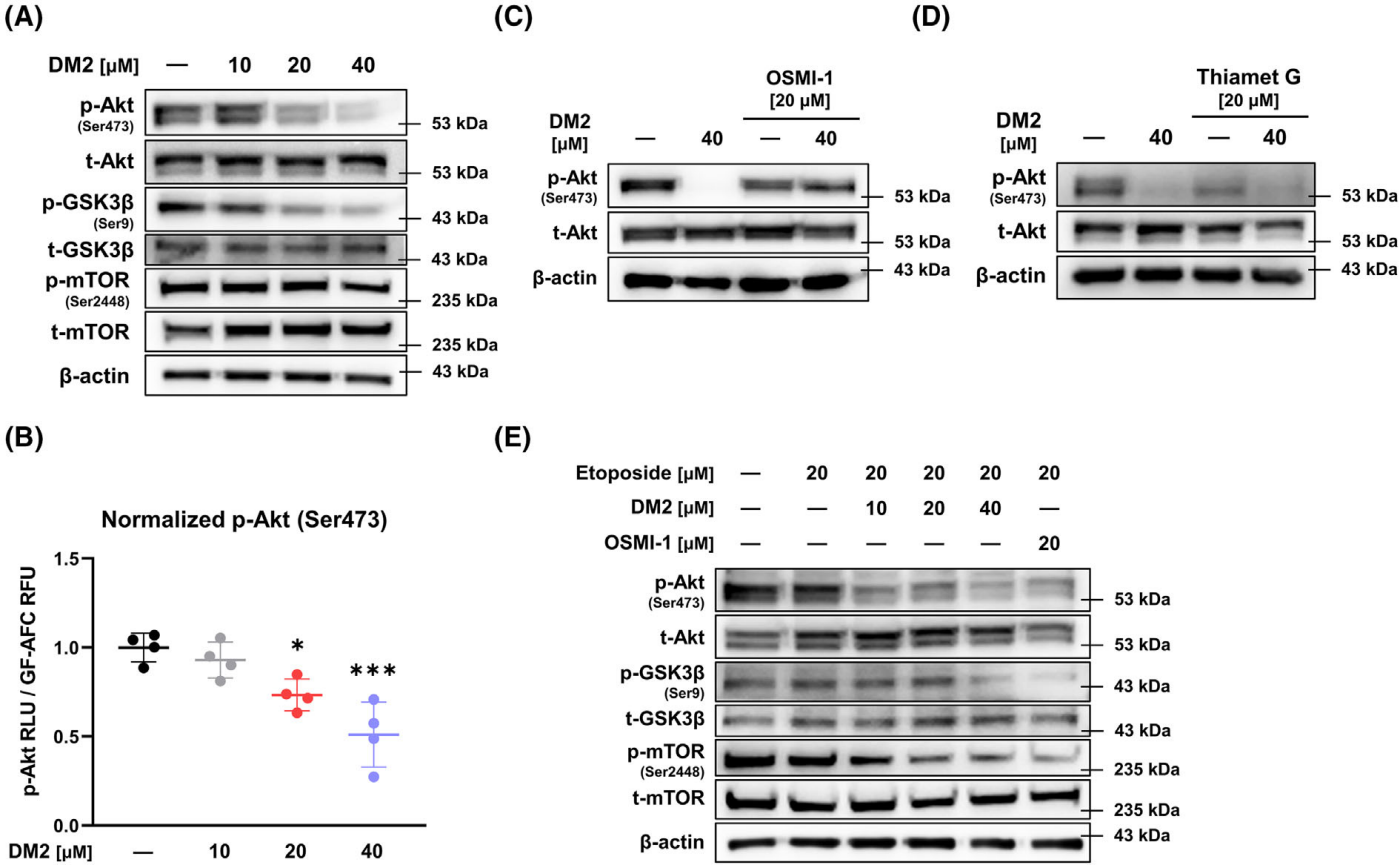
DM2 suppresses the Akt signaling pathway in a dose‐dependent manner. (A) Western blot analysis showing that 24 h of DM2 treatment dose‐dependently inhibits the phosphorylation of Akt (Ser473), GSK3β (Ser9), and mTOR (Ser2448) in HepG2 cells (*n* = 3). (B) Treatment with DM2 resulted in a significant reduction of p‐Akt (Ser473) luminescence (RLU) normalized to GF‐AFC fluorescence (RFU) (*n* = 4). (C) Cotreatment with 20 μm OSMI‐1 reduces Akt phosphorylation to a lesser extent than 40 μm DM2 treatment, and OSMI‐1 treatment reverses DM2‐mediated Akt inhibition (*n* = 3). (D) Thiamet G reduces Akt phosphorylation, with this reduction enhanced by DM2 cotreatment (*n* = 3). (E) DM2 dose‐dependently inhibits Akt signaling after 24 h in HepG2 cells treated with 20 μm etoposide and DM2 (*n* = 3). *P < 0.05 and ***P < 0.001 versus the control condition. Data represent means ± SD from at least three independent experiments. Statistical significance was determined with one‐way ANOVA with *post hoc* Tukey test.

To determine whether DM2 affects the Akt pathway, western blot analysis was conducted. Results showed that 24 h DM2 treatment dose‐dependently inhibited the phosphorylation of Akt (Ser473), GSK3β (Ser9), and mTOR (Ser2448) in HepG2 cells (Fig. 7A, Fig. S7A). Similarly, DM2 treatment led to a significant, dose‐dependent reduction in normalized p‐Akt (Ser473) levels (Fig. 7B, Fig. S7B). To further understand the relationship between *O*‐GlcNAc modulation and the Akt pathway, cells were co‐treated with OSMI‐1 or Thiamet G alongside DM2 for 24 h. Although 20 μm OSMI‐1 inhibited Akt phosphorylation, this effect was less pronounced than that of 40 μm DM2 (Fig. 7C, Fig. S7C). Interestingly, additional OSMI‐1 treatment partially restored the Akt phosphorylation inhibited by DM2. Thiamet G reduced Akt phosphorylation, and this effect was amplified through cotreatment with DM2 (Fig. 7D). In HepG2 cells treated with 20 μm etoposide, cotreatment with DM2 continued to inhibit the Akt pathway in a dose‐dependent manner (Fig. 7E). DM2 markedly inhibited Akt phosphorylation in Hep3B and A549 cells, highlighting its conserved ability to suppress the Akt signaling pathway across multiple cancer types (Fig. S8).

### 3.6 DM2–etoposide synergistic anticancer effect is mediated through the Akt signaling pathway

**Fig. 8:**
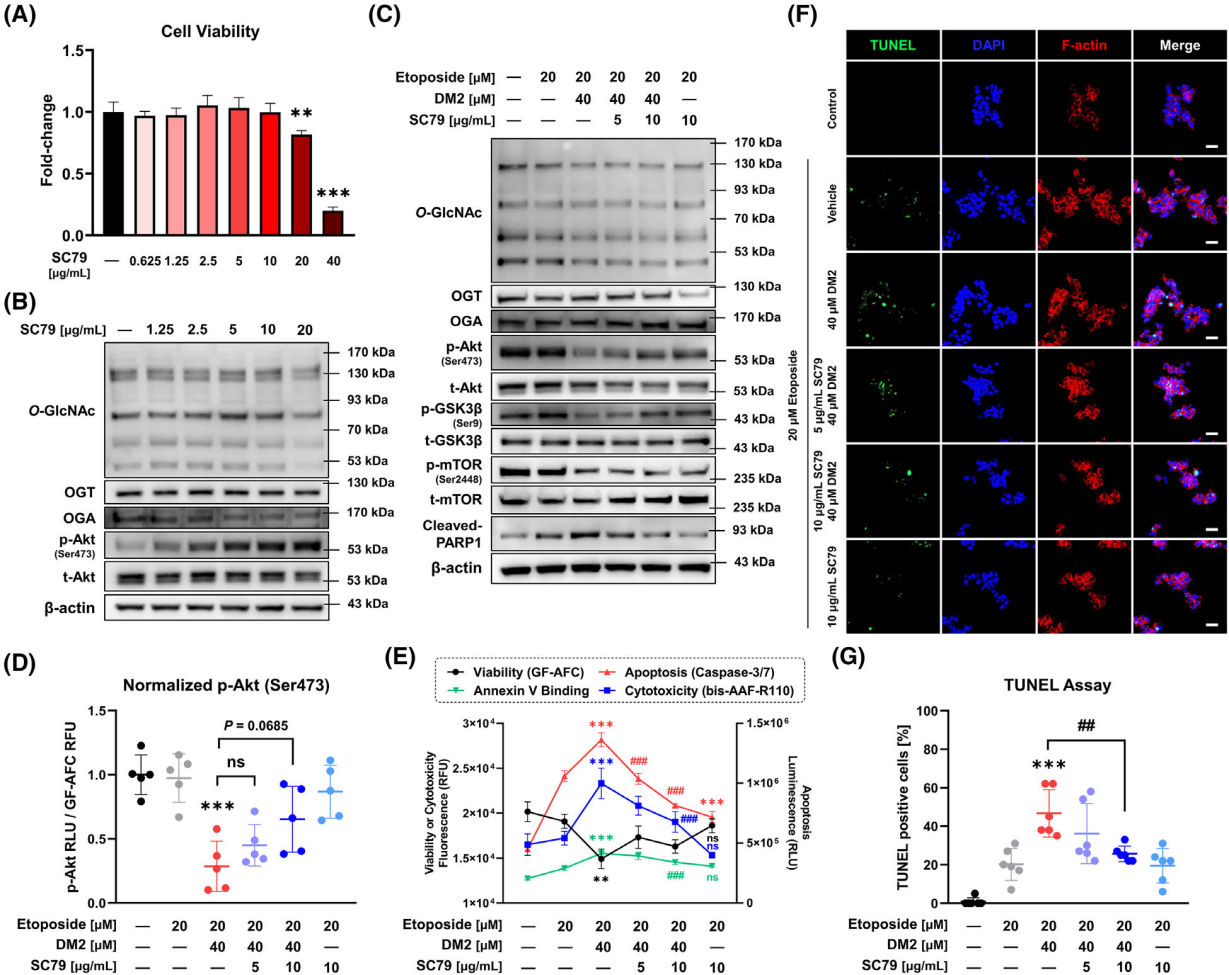
Synergistic anticancer effects of DM2 and etoposide are mediated through the Akt signaling pathway. Treatment with the Akt activator SC79 for 24 h does not affect HepG2 cell viability at concentrations up to 10 μm but reduces viability at 20 μm (*n* = 4). **P < 0.01 and ***P < 0.001 versus the control condition. (B) Western blot analysis confirming dose‐dependent increases in Akt phosphorylation upon SC79 treatment without effects on *O*‐GlcNAc levels (*n* = 3). (C) Cotreatment with SC79 does not restore the *O*‐GlcNAc reduction induced by etoposide and DM2 but dose‐dependently increases phosphorylation of Akt and GSK3β as well as inhibiting PARP1 cleavage (*n* = 3). (D) DM2‐etoposide cotreatment significantly decreased normalized p‐Akt (Ser473) levels, which were subsequently restored by SC79 (*n* = 5). (E) SC79 treatment restores GF‐AFC viability reduced by etoposide–DM2 cotreatment (*n* = 3) and dose‐dependently inhibits bis‐AAF‐R110 cytotoxicity (*n* = 4), caspase‐3/7 activity (*n* = 3), and Annexin V binding (*n* = 4). (F, G) TUNEL assay results confirming that DM2's synergistic anticancer effects with etoposide are mediated through the Akt pathway (*n* = 6; 200×; white scale bar: 20 μm). **P < 0.01 and ***P < 0.001 versus the etoposide‐only treatment group; ^##^ *P* < 0.01 and ^###^ *P* < 0.001 versus the etoposide–DM2 cotreatment group. Data represent means ± SD from at least three independent experiments. Statistical significance was determined with one‐way ANOVA with *post hoc* Tukey test. ns, not significant.

To confirm whether the synergistic effect of DM2 operates through the Akt pathway, additional studies were conducted using SC79, an Akt activator. SC79 had no effect on cell viability in HepG2 cells at concentrations up to 10 μg·mL^−1^, but viability decreased at 20 μg·mL^−1^ (Fig. 8A). Notably, at non‐cytotoxic concentrations, SC79 dose‐dependently increased Akt phosphorylation without altering *O*‐GlcNAc levels (Fig. 8B, Fig. S9). Notably, the DM2–etoposide cotreatment–induced reduction in *O*‐GlcNAc levels was not restored by SC79 (Fig. 8C, Fig. S10). However, SC79 treatment increased Akt and GSK3β phosphorylation in a dose‐dependent manner. Furthermore, SC79 treatment inhibited PARP1 cleavage induced by etoposide and DM2, suggesting that the synergistic anticancer effects of DM2 are mediated through the Akt pathway. Consistent with these findings, DM2–etoposide cotreatment significantly decreased normalized p‐Akt (Ser473) levels, which, although not significantly restored, tended to recover upon SC79 treatment (Fig. 8D). Moreover, SC79 restored GF‐AFC viability decreased by DM2–etoposide cotreatment (Fig. 8E). SC79 also dose‐dependently inhibited bis‐AAF‐R110 cytotoxicity, caspase‐3/7 activity, and Annexin V binding. This was corroborated by TUNEL assay results (Fig. 8F,G), which confirmed that DM2's synergistic effect is primarily achieved via the Akt pathway.

## 4 Discussion

DM2, a precursor to ginsenosides, has been underexplored compared with its well‐studied derivatives. The present study demonstrated that DM2 directly interacts with OGT, leading to downregulation of *O*‐GlcNAcylation (Figs 1D, 2F, 6A). Using Pharmaco‐Net, a deep learning–based computational platform, we confirmed this interaction (Fig. 2A–E). Pharmaco‐Net represents a transformative approach to evaluating and predicting drug efficacy, toxicity, and mechanisms by leveraging neural networks trained on extensive datasets [24, 25]. This methodology effectively models complex molecular and cellular interactions between chemotherapeutic drugs and biological systems. One notable example is its role in identifying baricitinib as an inhibitor of adaptor protein complex 2, which is involved in the infection of host cells by severe acute respiratory syndrome coronavirus 2 [26]. Baricitinib subsequently progressed to clinical trials and received Emergency Use Authorization from the US Food and Drug Administration for COVID‐19 treatment [27, 28]. Pharmaco‐Net's predictive accuracy was validated by comparing its binding energy and affinity values for OSMI‐1, a well‐characterized OGT inhibitor (IC_50_: 2.7 μm) [29, 30], to empirical data (Table 2). OSMI‐1's predicted binding energy (−6.36 kcal·mol^−1^) and affinity (0.0203 μm) aligned closely with its observed inhibitory potency. Similarly, the DM2–OGT interaction identified via the Pharmaco‐Net platform was corroborated through an OGT activity assay (Fig. 2F, Table 2), underscoring the platform's utility in drug evaluation and early‐stage drug screening. Interestingly, ginsenoside Rb1, a derivative of DM2, has been shown to mitigate diabetic cardiomyopathy *in vivo* by diminishing protein *O*‐GlcNAcylation and enhancing sarcoplasmic reticulum Ca^2+^‐ATPase 2a expression and activity [31]. Future investigations should delineate the conserved structural motifs conserved between DM2 and its derivatives to identify features critical for OGT inhibition. Deep learning–based platforms, such as Pharmaco‐Net, provide a highly effective framework for systematically identifying and optimizing these inhibitory features.

Although both DM2 and OSMI‐1 reduced *O*‐GlcNAc levels, their effects differed across protein size fractions (Fig. 1F). *O*‐GlcNAcylation is a dynamic process governed by tightly regulated feedback mechanisms that maintain cellular homeostasis. For example, Thiamet G–induced *O*‐GlcNAc upregulation is associated with enhanced OGA transcription via *O*‐GlcNAc‐dependent regulatory mechanisms in SH‐SY5Y human neuroblastoma cells [32]. Similarly, OSMI‐1 treatment has been shown to upregulate and downregulate OGT and OGA protein levels, respectively, in NCI‐H508 cells [30]. Aligning with these findings, we observed that OGT levels increased following OSMI‐1 treatment, as previously reported, but decreased upon DM2 treatment. These contrasting patterns suggest DM2's mechanism of action differs from that of OSMI‐1 and may involve novel pathways for OGT interaction. We also observed that the regulatory patterns of OGT protein and mRNA expression did not align following treatment with DM2 and OSMI‐1 (Figs S2, S4, S5). Notably, DM2 treatment significantly reduced both *O*‐GlcNAc levels and OGT protein expression, whereas it markedly increased OGT mRNA expression. *Moreover*, DM2 significantly upregulated OGT mRNA levels when co‐treated with OSMI‐1, Thiamet G, and etoposide. The discordance between OGT protein and mRNA levels following DM2 treatment suggests the involvement of post‐transcriptional or translational regulatory mechanisms. Previous studies have shown that OGT can be subject to rapid turnover depending on cellular stress or metabolic status, despite elevated transcript levels [33]. This uncoupling of mRNA and protein abundance has also been reported under pharmacological perturbation of *O*‐GlcNAc cycling enzymes, reflecting compensatory homeostatic feedback to maintain intracellular *O*‐GlcNAc balance [34, 35]. Therefore, the observed increase in OGT mRNA upon DM2 treatment may represent a compensatory transcriptional response to reduced *O*‐GlcNAc levels, while the concurrent decrease in OGT protein suggests that DM2 may promote post‐translational degradation of OGT or inhibit its translation. This pattern underscores the distinct mechanism of DM2 compared to canonical OGT inhibitors such as OSMI‐1, potentially implicating DM2 in the modulation of upstream signaling or proteostatic pathways that regulate OGT stability. Combined treatment with etoposide, DM2, and OSMI‐1 showed a slight but not statistically significant reduction in cell viability compared to etoposide and DM2 treatment alone (Fig. S11A). Interestingly, detailed prediction results obtained using Pharmaco‐Net revealed that DM2 and OSMI‐1 bind to the same binding site but at distinct positional orientations (Fig. S11B). This implies that the two compounds may exhibit partial competitive binding, or alternatively, induce subtle conformational changes in the binding microenvironment that modulate OGT activity indirectly. The distinct changes in OGT expression induced by DM2 and OSMI‐1 treatment indirectly reflect the complex regulatory mechanisms governing OGT (Fig. S3B,E). Taken together, the observed tendency for further reduction in cell viability upon combined treatment with etoposide, DM2, and OSMI‐1 is likely attributable to these micro‐level alterations in binding site interactions. Moreover, these findings raise the intriguing possibility that a multi‐ligand occupancy mechanism within the same catalytic site may underlie the complex regulation of OGT activity. Future work should aim to experimentally validate these *in silico* predictions by assessing the binding affinity and kinetics of OGT with each inhibitor in *in vitro* settings, to delineate how such positional differences translate into functional modulation of enzymatic activity.

*O*‐GlcNAcylation is implicated in critical oncogenic processes, including tumor progression, metastasis, and cancer‐associated inflammation, through the regulation of key processes, such as epithelial–mesenchymal transition, cancer cell metabolism, and NF‐κB activity [36, 37]. Prior studies have shown that OGT inhibition reduces cancer cell proliferation, migration, and invasion by disrupting these pathways and destabilizing key oncogenic proteins, for example, enhancer of zeste homolog 2 [38, 39]. Consistent with these findings, our results indicated that DM2 significantly inhibited Akt activity through decreased phosphorylation at Ser473 in HepG2, Hep3B, and A549 cells (Fig. 7A, Fig. S8). Given that the PI3K/Akt signaling pathway is central to tumor initiation and progression [40, 41], DM2's ability to attenuate this pathway further underscores its potential as an adjunct to existing anticancer therapies.

As an anticancer agent, etoposide has several limitations, including the development of drug resistance through Topo II mutations, adverse effects (e.g., myelosuppression and gastrointestinal toxicity), and risks of secondary malignancies, such as treatment‐related leukemia [42]. In the present study, etoposide concentrations up to 50 μm had no significant impact on HepG2 cell viability over 24 h, as reflected by the observed IC_50_ value of 105.61 μm (Fig. 3D,E). These findings underscore the need for improved drug delivery systems, combination therapies, and next‐generation Topo II inhibitors to improve therapeutic outcomes.

Our previous studies revealed that the reduction of *O*‐GlcNAcylation through the OGT inhibitor OSMI‐1 enhanced the apoptosis‐inducing effects of etoposide [23], suggesting that compounds downregulating *O*‐GlcNAc levels may amplify the anticancer effects of Topo II inhibitors, including etoposide and doxorubicin. Although etoposide has also been reported to reduce O‐GlcNAcylation [43], the underlying molecular mechanism remains unclear. Interestingly, we found that treatment with etoposide for 24 h significantly upregulated both OGT and OGA expression (Fig. S5). The increase in OGT and OGA mRNA expression suggests the possibility of a compensatory feedback regulation in response to the reduction in *O*‐GlcNAc levels. A decrease in *O*‐GlcNAc may induce a transcriptional response aimed at upregulating OGT expression to maintain cellular homeostasis, while OGA expression may also be coordinately regulated to preserve the balancing kinetics of *O*‐GlcNAc cycling. Etoposide‐induced DNA damage promotes a relocalization of OGT and results in substrate‐selective remodeling of *O*‐GlcNAcylation patterns, while stress‐associated metabolic reprogramming—including alterations in the hexosamine biosynthetic pathway (HBP) and AMPK activation—can reduce overall UDP‐GlcNAc availability, thereby lowering global *O*‐GlcNAcylation levels [44, 45, 46]. These mechanisms are consistent with reports of overall *O*‐GlcNAc reduction after etoposide treatment and provide a rationale for its synergy with DM2. In this context, quantifying intracellular UDP‐GlcNAc levels could provide mechanistic insight into how both etoposide and DM2 modulate *O*‐GlcNAcylation. For instance, in a related observation, although Thiamet G reduced OGA protein levels, global *O*‐GlcNAcylation decreased, likely due to metabolic stress–induced reduction of UDP‐GlcNAc availability and substrate‐selective *O*‐GlcNAc remodeling, rather than changes in OGA abundance alone [47, 48]. This result highlights that *O*‐GlcNAc homeostasis is predominantly regulated by enzymatic activity and metabolic flux, rather than OGT and OGA expression levels.

Although DM2 exhibited no cytotoxicity in HepG2 cells within 24 h of standalone treatment, its combination with etoposide significantly enhanced apoptosis‐mediated cytotoxicity (Figs 1C, 5C). Interestingly, treatment with DM2 at concentrations of 10 and 20 μm for 24 h increased the cell viability of HepG2 cells. Further investigations are required to determine whether this effect is attributable to an off‐target action of DM2 or represents a secondary, context‐dependent cellular response. Notably, DM2 reduced the IC_50_ value of etoposide for cell viability by 2.29‐fold and the EC_50_ value for caspase‐3/7 activity by 3.64‐fold (Figs 3E, 5D). These effects became more pronounced over time (Fig. 5B), as evidenced by a 2.56‐fold increase in Annexin V luminescence and a 4.42‐fold increase in cytotoxicity fluorescence when HepG2 cells were treated with DM2 and 2.5 μm etoposide for 36 h (Fig. 5C; bottom panel). The observed synergy can be attributed to the modulation of key apoptotic pathways. PARP1, p53, and caspase‐3 have well‐established roles in promoting apoptosis and enhancing the efficacy of anticancer therapies [49]. Targeting PARP1, particularly in cancers with p53 mutations or deficiencies, has shown promise for inducing synthetic lethality [50]. Modulating these pathways using small molecules or inhibitors, such as veliparib and diosgenin, has also been reported to enhance apoptosis [51, 52]. Our findings showed that etoposide at 20 μm did not independently affect p53 and cleaved‐PARP1 levels; however, their expression significantly increased in the presence of DM2 within 24 h (Fig. 5E). Importantly, the observed anticancer synergy between DM2 and etoposide in HepG2 cells was largely conserved across additional liver cancer cell lines (Fig. 6B). However, the time‐dependent enhancement of cytotoxicity observed selectively in Huh7 and SK‐HEP1 cells upon 36 h of cotreatment (Fig. 6C) raises the possibility of variable apoptotic thresholds or compensatory survival pathways among different tumor types. These differences may inform the identification of predictive biomarkers for therapeutic responsiveness and support further investigation of DM2 in combinatorial treatment regimens.

In this study, we demonstrated that DM2 enhances the anticancer activity of etoposide by modulating *O*‐GlcNAcylation and promoting apoptotic signaling in multiple HCC cell lines. The synergistic cytotoxic effects consistently observed in HepG2, Hep3B, Huh7, SK‐HEP1, SNU‐182, and SNU‐387 cells indicate that the DM2–etoposide combination possesses broad therapeutic potential against HCC (Figs 3C, 6B, S6B). Considering the pivotal role of *O*‐GlcNAc modification in tumor cell metabolism and survival, it would be important for future investigations to determine whether HCCs with inherently high *O*‐GlcNAc levels exhibit greater sensitivity to this combination treatment. Glypican‐3 (GPC3), a membrane‐bound heparan sulfate proteoglycan, functions as an oncofetal antigen and represents a well‐established diagnostic and therapeutic biomarker for HCC [53, 54]. It is abundantly expressed in most HCC but absent in normal adult liver tissue, making it an attractive target for antibody‐based therapies and drug delivery systems [55]. Notably, HepG2, Huh7, Hep3B, SNU‐182, and SNU‐387 cells are reported to be GPC3‐positive [56, 57, 58], supporting their use as representative models of GPC3‐expressing HCC. Given that both *O*‐GlcNAcylation and GPC3 signaling are implicated in HCC progression and treatment resistance, future studies should explore the potential interplay between *O*‐GlcNAc levels and GPC3 expression in determining therapeutic responsiveness. Specifically, it would be of great interest to investigate whether GPC3 could serve as a therapeutic response biomarker for DM2–etoposide combination therapy. Elucidating this relationship may provide mechanistic insights into how glycosylation dynamics and oncofetal antigen expression jointly regulate apoptotic sensitivity in hepatocellular carcinoma and could facilitate the stratification of patient subgroups most likely to benefit from this combination regimens.

Excessive Akt activation drives tumor development by suppressing apoptotic pathways, promoting cellular growth, and contributing to resistance against chemotherapeutic agents. [59]. We found that the etoposide–DM2 combination significantly suppressed Akt activity, providing a mechanistic basis for their synergistic anticancer effects in HepG2 cells (Fig. 7F). By targeting Akt, this combination therapy may not only enhance apoptotic responses but also sensitize cancer cells to chemotherapy, highlighting its therapeutic potential in liver cancer treatment. Elevated *O*‐GlcNAcylation levels have been shown to enhance Akt activity and cellular invasiveness in gastric cancer cells [60]. Similarly, a 24‐h treatment with OSMI‐1 led to reduced Akt activity in HepG2 cells; however, Akt activity decreased following Thiamet G treatment (Fig. 7C,D). A prior study in mouse vascular smooth muscle cells indicated that enhanced *O*‐GlcNAcylation, induced by shOGA treatment, transiently increased p‐Akt (Ser473) expression levels (within 10 min) before a subsequent decline, indicating dynamic regulation of Akt activity by *O*‐GlcNAcylation [61]. Notably, the decrease in p‐Akt induced by DM2 was restored upon OSMI‐1 treatment (Fig. 7C and S7B). One hypothesis is that DM2, a relatively uncharacterized dammarane triterpenoid, exerts off‐target effects beyond OGT inhibition, leading to a stronger suppression of p‐Akt than OSMI‐1. Previous studies have reported that DM2 modulates VEGF‐induced ROS generation in endothelial cells, suggesting that its effect on Akt phosphorylation may involve broader mechanisms than OGT inhibition alone. Furthermore, DM2 and OSMI‐1 exhibited distinct patterns of *O*‐GlcNAc reduction (Fig. 1F), implying that each inhibitor interacts with OGT through different mechanisms. These differences may underlie the paradoxical elevation of p‐Akt observed during cotreatment, despite each compound individually reducing it.

The use of the Akt activator SC79 provided additional insights into the interplay between *O*‐GlcNAcylation and Akt signaling. SC79, known to prevent hepatocyte apoptosis [62], attenuated the elevated cleaved‐PARP1 levels and caspase‐3/7 activity induced by etoposide–DM2 cotreatment (Fig. 8C,E), confirming that their synergistic effect is mediated through the Akt pathway. Notably, SC79 treatment at non‐cytotoxic concentrations alone did not appear to directly affect *O*‐GlcNAc levels; however, it restored p‐Akt (Ser473) levels in cells treated with etoposide and DM2 (Fig. 8B–D, Figs S9, S10), suggesting that Akt signaling operates downstream of etoposide–DM2‐mediated *O*‐GlcNAcylation regulation. The decrease in *O*‐GlcNAc levels observed at a concentration of 20 μg·mL^−1^ SC79 may be attributable to cytotoxic effects induced by SC79 itself. Additional experiments are required to clearly determine this possibility.

Although both DM2 and OSMI‐1 reduced *O*‐GlcNAc levels in HepG2 cells, DM2 exhibited a stronger inhibitory effect on Akt phosphorylation, indicating that additional pathways may influence Akt activity (Figs 1F and 7B). Previous studies have shown that *O*‐GlcNAcylation and mTOR signaling are cross‐regulated [63], with elevated *O*‐GlcNAcylation enhancing mTOR activity in cancer cells, and mTOR stabilizing OGT to promote *O*‐GlcNAcylation. Our findings demonstrated that the etoposide–DM2 combination effectively inhibits mTOR signaling, a downstream effector of Akt, thereby reducing cancer cell survival (Figs 7F and 8E). Thus, the combination of etoposide and DM2 effectively suppresses Akt activity, likely through *O*‐GlcNAcylation regulation, enhancing apoptotic responses and impairing cancer cell survival through p53‐mediated signaling.

Despite these promising results, several limitations must be addressed to strengthen the translational potential of our findings. First, our study focused on cellular and molecular analyses; *in vivo* experiments are required to confirm the reproducibility of these effects and establish the optimal drug combination ratios for maximizing synergistic efficacy while minimizing side effects. Additionally, we did not include experiments using normal healthy liver cell lines, indicating the need for further validation in non‐cancerous hepatocytes. Because *O*‐GlcNAcylation *in vivo* is dynamically regulated by OGT and OGA, the DM2‐induced reduction of *O*‐GlcNAc levels observed in HepG2 cells may result, at least in part, from increased OGA activity acting either directly or indirectly. AI‐based prediction analysis using Pharmaco‐Net suggested a low probability of direct interaction between DM2 and OGA. However, it remains possible that DM2 may influence OGA activity through indirect mechanisms or upstream signaling pathways, thereby amplifying its overall effect on *O*‐GlcNAc turnover. Further studies will be required to clarify the potential involvement of OGA in this process. Additionally, the mechanism underlying etoposide‐induced *O*‐GlcNAcylation reduction remains unclear, and elucidating whether this reduction is integral to its anticancer action or a downstream consequence would provide deeper insights into DM2's mechanisms of action. Finally, although our preliminary results suggest synergistic potential in lung and colon cancer cells (Fig. S6B), further research is needed to identify the cancer types most suitable for clinical application. Addressing these gaps will be crucial for advancing DM2 as a therapeutic agent and translating these findings into effective cancer treatments.

## 5 Conclusions

In conclusion, our study underscores the potential of DM2 to enhance the sensitivity of liver cancer cells to etoposide by reducing *O*‐GlcNAcylation and Akt activity. These findings highlight the unique mechanism of action of DM2 relative to other OGT inhibitors, offering promise for its integration into combination therapies aimed at overcoming drug resistance and improving anticancer efficacy. However, further research is needed to elucidate the molecular mechanisms underlying DM2's effects, optimize drug delivery and combination strategies, and validate its therapeutic potential *in vivo* and across different cancer types. By establishing a foundation for understanding DM2's role as an adjuvant in cancer treatment, this study opens new opportunities for its application in enhancing the efficacy of chemotherapeutic regimens beyond liver cancer cells.

## Conflict of interest

Authors (JL, BH, GK, JP, MK, and SL) are employees of Korea Ginseng Corporation, which funded this research. However, the company had no influence on the study design, data interpretation, or publication decision.

## Author contributions

JL, GK, and BH designed the experiments under the supervision of SL and SR. JL wrote the manuscript. JL, JP, MK, YP, and JC performed the experiments and analyzed the data. JL, GK, and BH performed literature research. JL designed the research template. All authors have read and agreed to the published version of the manuscript.

## Ethics statement

This study did not include research involving human participants or animal experimentation.

## Supporting information


**Fig. S1.** Quantitative evaluation of band intensities in the Fig. 1D western blot analysis.
**Fig. S2.** Effects of treatment with DM2 on OGT and OGA mRNA expression in HepG2 cells.
**Fig. S3.** Quantitative evaluation of band intensities in the Fig. 1F western blot analysis.
**Fig. S4.** Effects of single and combined treatment with DM2, OSMI‐1, and Thiamet‐G on OGT and OGA mRNA expression in HepG2 cells.
**Fig. S5.** Effects of single and combined treatment with etoposide, DM2, and OSMI‐1 on OGT and OGA mRNA expression in HepG2 cells.
**Fig. S6.** DM2 reduces O‐GlcNAc levels and enhances etoposide‐induced cytotoxicity and across various human cancer cell lines.
**Fig. S7.** Quantitative evaluation of band intensities in the Fig. 7A–C western blot analysis.
**Fig. S8.** Differential modulation of Akt by DM2 across cancer cell lines.
**Fig. S9.** Quantitative evaluation of band intensities in the Fig. 8B western blot analysis.
**Fig. S10.** Quantitative evaluation of band intensities in the Fig. 8C western blot analysis.
**Fig. S11.** DM2 and OSMI‐1 exhibit anticancer synergy through distinct binding orientations at the same OGT site.

**Table S1.** Primer sequences used for RT‐qPCR.

## Data Availability

The data that support the findings of this study are available from the corresponding author upon reasonable request.
