# Supplementary material for: Dammarenediol II enhances etoposide‐induced apoptosis by targeting O‐GlcNAc transferase and Akt/GSK3β/mTOR signaling in liver cancer
Source: Mol Oncol. 2025 Dec 30;20(6):1591–611. doi: 10.1002/1878-0261.70199 (PMC13238812; doi:10.1002/1878-0261.70199)
Supplement: Supplementary file 1 — Fig. S1. Quantitative evaluation of band intensities in the Fig. 1D western blot analysis. Fig. S2. Effects of treatment with DM2 on OGT and OGA mRNA expression in HepG2 cells. Fig. S3. Quantitative evaluation of band intensities in the Fig. 1F western blot analysis. Fig. S4. Effects of single and combined treatment with DM2, OSMI‐1, and Thiamet‐G on OGT and OGA mRNA expression in HepG2 cells. Fig. S5. Effects of single and combined treatment with etoposide, DM2, and OSMI‐1 on OGT and OGA mRNA expression in HepG2 cells. Fig. S6. DM2 reduces O‐GlcNAc levels and enhances etoposide‐induced cytotoxicity and across various human cancer cell lines. Fig. S7. Quantitative evaluation of band intensities in the Fig. 7A–C western blot analysis. Fig. S8. Differential modulation of Akt by DM2 across cancer cell lines. Fig. S9. Quantitative evaluation of band intensities in the Fig. 8B western blot analysis. Fig. S10. Quantitative evaluation of band intensities in the Fig. 8C western blot analysis. Fig. S11. DM2 and OSMI‐1 exhibit anticancer synergy through distinct binding orientations at the same OGT site. [file MOL2-20-1591-s001.docx]

Supplementary Figures


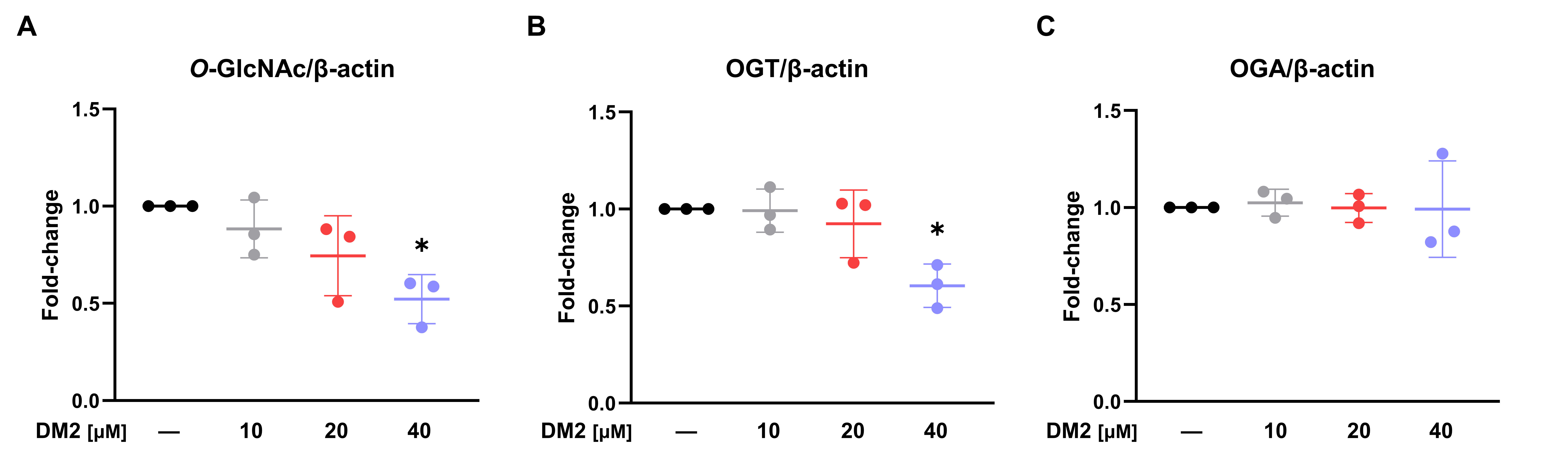


**Fig. S1. Quantitative evaluation of band intensities in the Fig. 1D western blot analysis.**

Densitometry (ImageJ) was performed on the bands shown in Fig. 1D. Band intensities were normalized to the loading control (β-actin) and then expressed relative to the matched control within each experiment (control = 1.0 in every replicate). (A) *O*-GlcNAc (*n* = 3), (B) OGT (*n* = 3), (C) OGA (*n* = 3). * *P* < 0.05 versus the control condition. Data represent means ± SD from three independent experiments. Statistical significance was determined with one-way repeated-measures ANOVA with post hoc Tukey test.


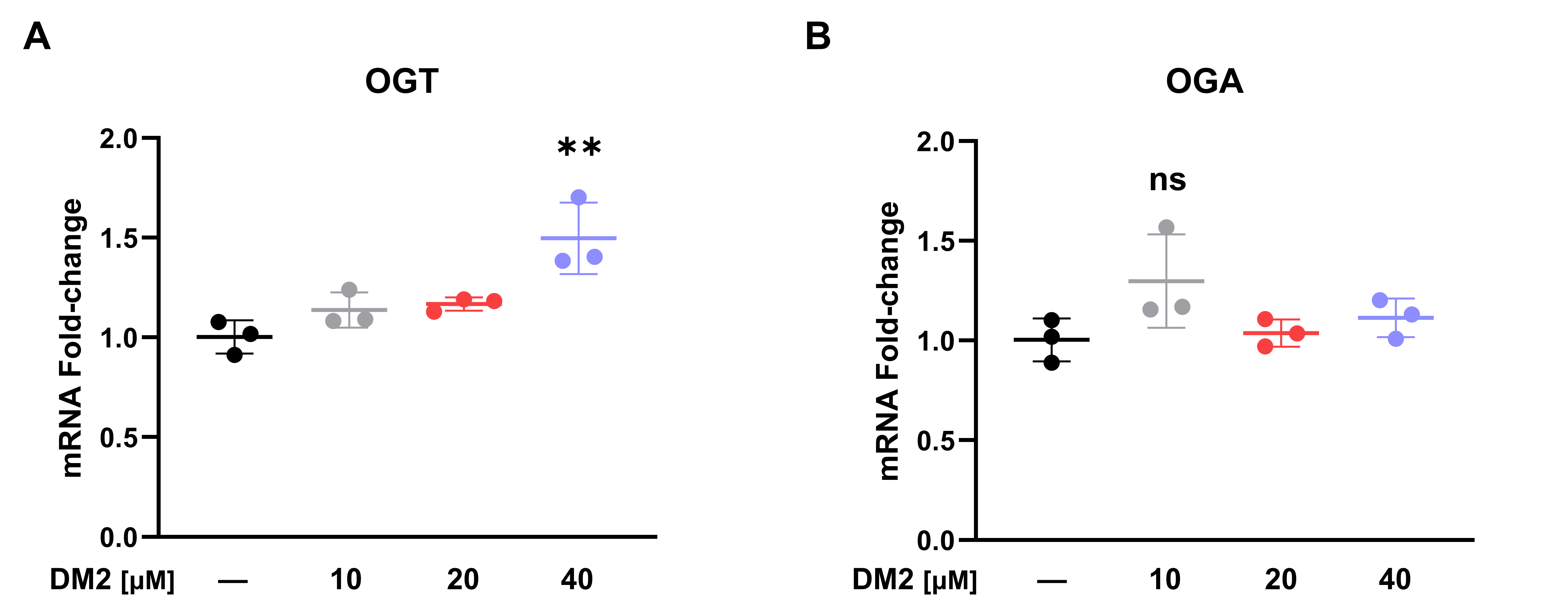


**Fig. S2. Effects of treatment with DM2 on OGT and OGA mRNA expression in HepG2 cells.**

HepG2 cells were treated with DM2 for 24 h. Total RNA was isolated, and RT-qPCR was performed using gene-specific primers. Relative expression levels were calculated using the ΔΔCt method and normalized to GAPDH. (A) OGT mRNA expression is significantly increased in HepG2 cells following 24 h treatment with 40 μM DM2 compared to the untreated control. (B) In contrast, OGA mRNA expression shows no significant changes across all treatment conditions (*n* = 3). ** *P* < 0.01 versus the control condition. Data represent means ± SD from three independent experiments. Statistical significance was determined with one-way repeated-measures ANOVA with post hoc Tukey test. ns, not significant.


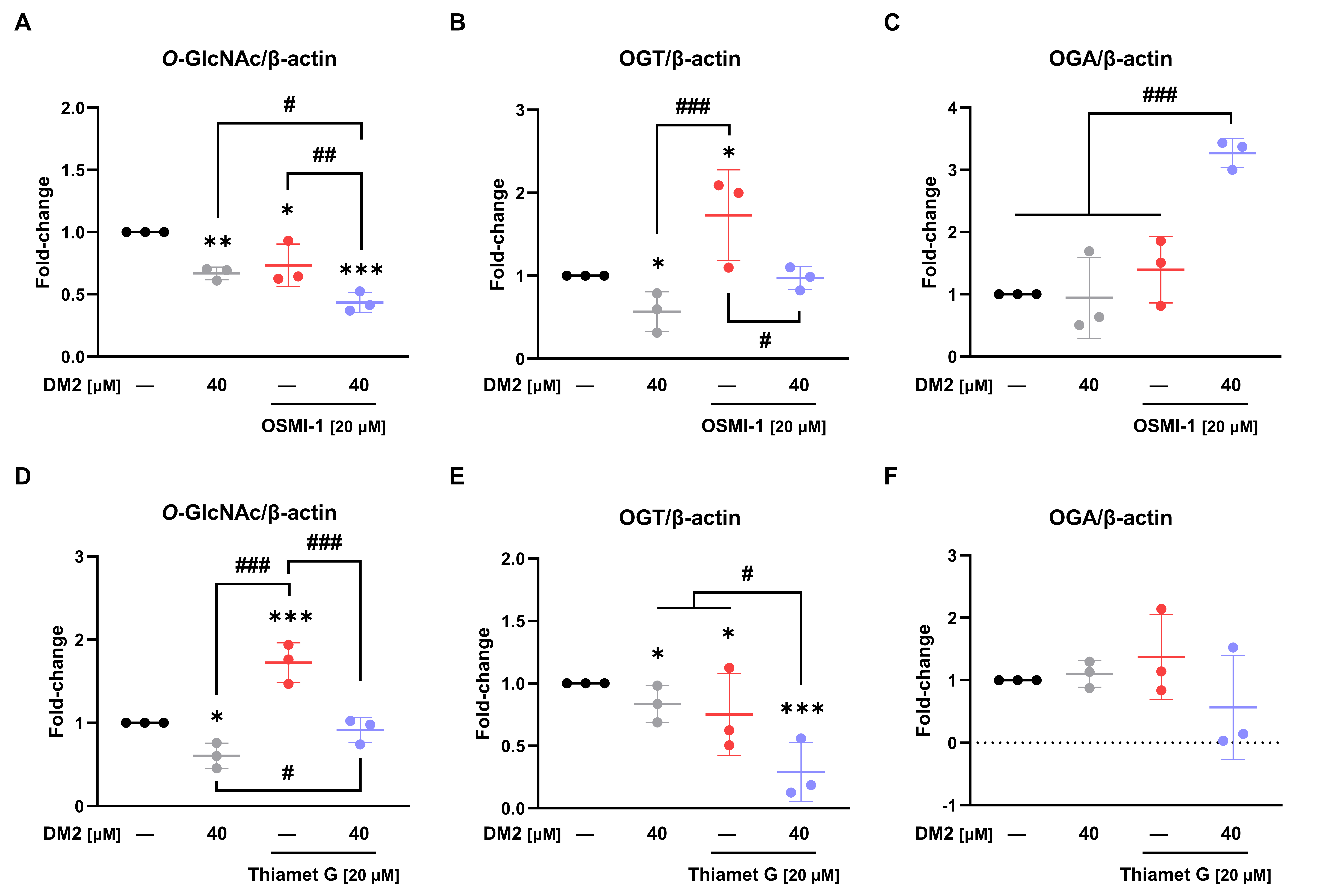


**Fig. S3. Quantitative evaluation of band intensities in the Fig. 1F western blot analysis.**

Densitometry (ImageJ) was performed on the bands shown in Fig. 1F. Band intensities were normalized to the loading control (β-actin) and then expressed relative to the matched control within each experiment (control = 1.0 in every replicate). Panels are organized by drug inclusion: (A–C) datasets including the OSMI-1-treated condition—(A) O-GlcNAc (*n* = 3), (B) OGT (*n* = 3), (C) OGA (*n* = 3); (D–F) datasets including the Thiamet G-treated condition—(A) *O*-GlcNAc (*n* = 3), (B) OGT (*n* = 3), (C) OGA (*n* = 3). * *P* < 0.05, ** *P* < 0.01, and *** *P* < 0.001 versus the control condition; # *P* < 0.05, ## *P* < 0.01, ### *P* < 0.001 for pairwise comparisons between matched conditions as indicated by connecting lines on the graph. Data represent means ± SD from three independent experiments. Statistical significance was determined with two-way repeated-measures ANOVA with post hoc Tukey test.


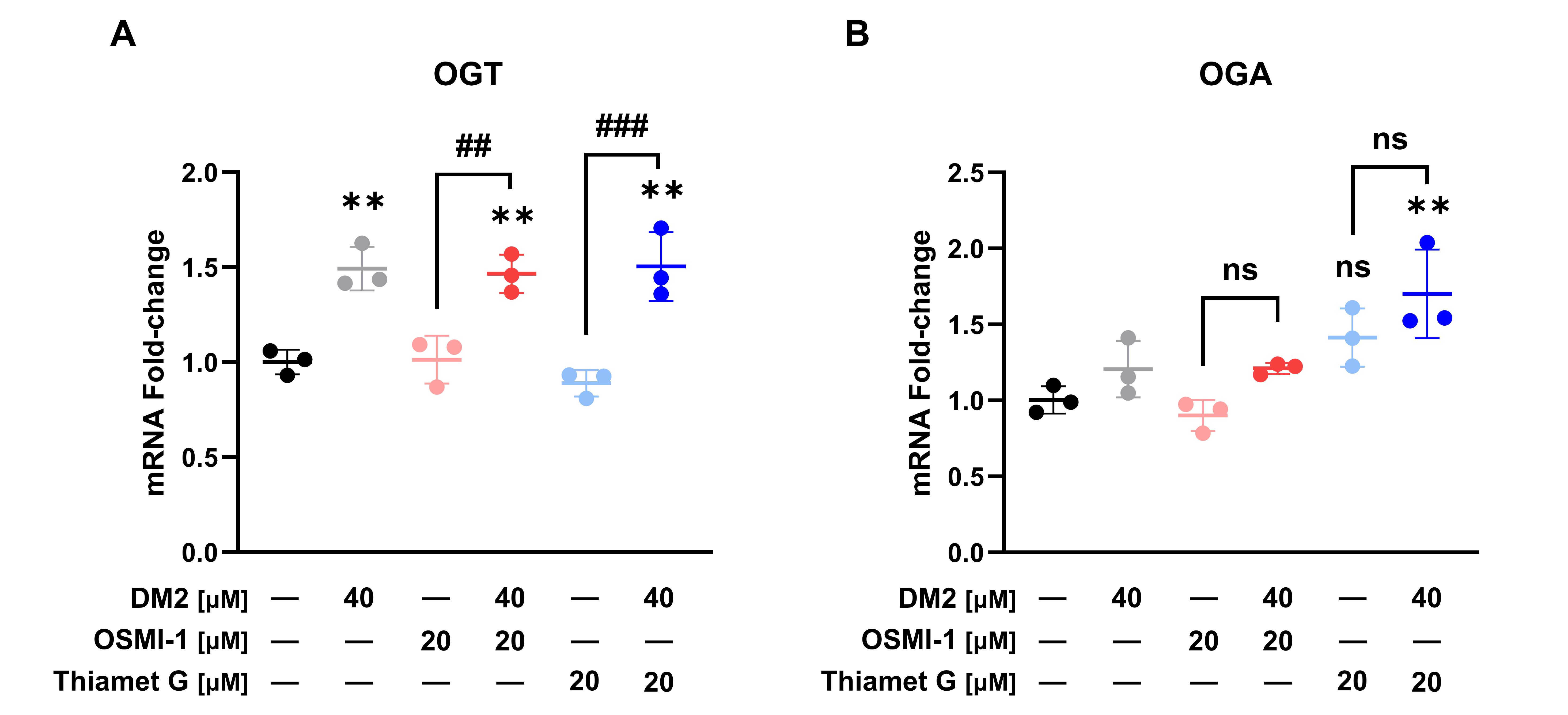


**Fig. S4. Effects of single and combined treatment with DM2, OSMI-1, and Thiamet-G on OGT and OGA mRNA expression in HepG2 cells.**

HepG2 cells were treated with the indicated compounds for 24 h. Total RNA was isolated, and RT-qPCR was performed using gene-specific primers. Relative expression levels were calculated using the ΔΔCt method and normalized to GAPDH. (A) DM2 treatment significantly increases OGT mRNA expression, whereas OSMI-1 or Thiamet-G alone does not alter OGT levels. However, cotreatment of DM2 with either OSMI-1 or Thiamet-G results in a significant increase in OGT mRNA expression compared to OSMI-1 or Thiamet-G treatment alone. (B) Among the tested conditions, only the combined treatment of DM2 and Thiamet-G lead to a significant increase in OGA mRNA expression relative to the control (*n* = 3). ** *P* < 0.01 versus the control condition. ## *P* < 0.01 and ### *P* < 0.001 for pairwise comparisons between matched conditions as indicated by connecting lines on the graph. Data represent means ± SD from three independent experiments. Statistical significance was determined with one-way repeated-measures ANOVA with post hoc Tukey test. ns, not significant.


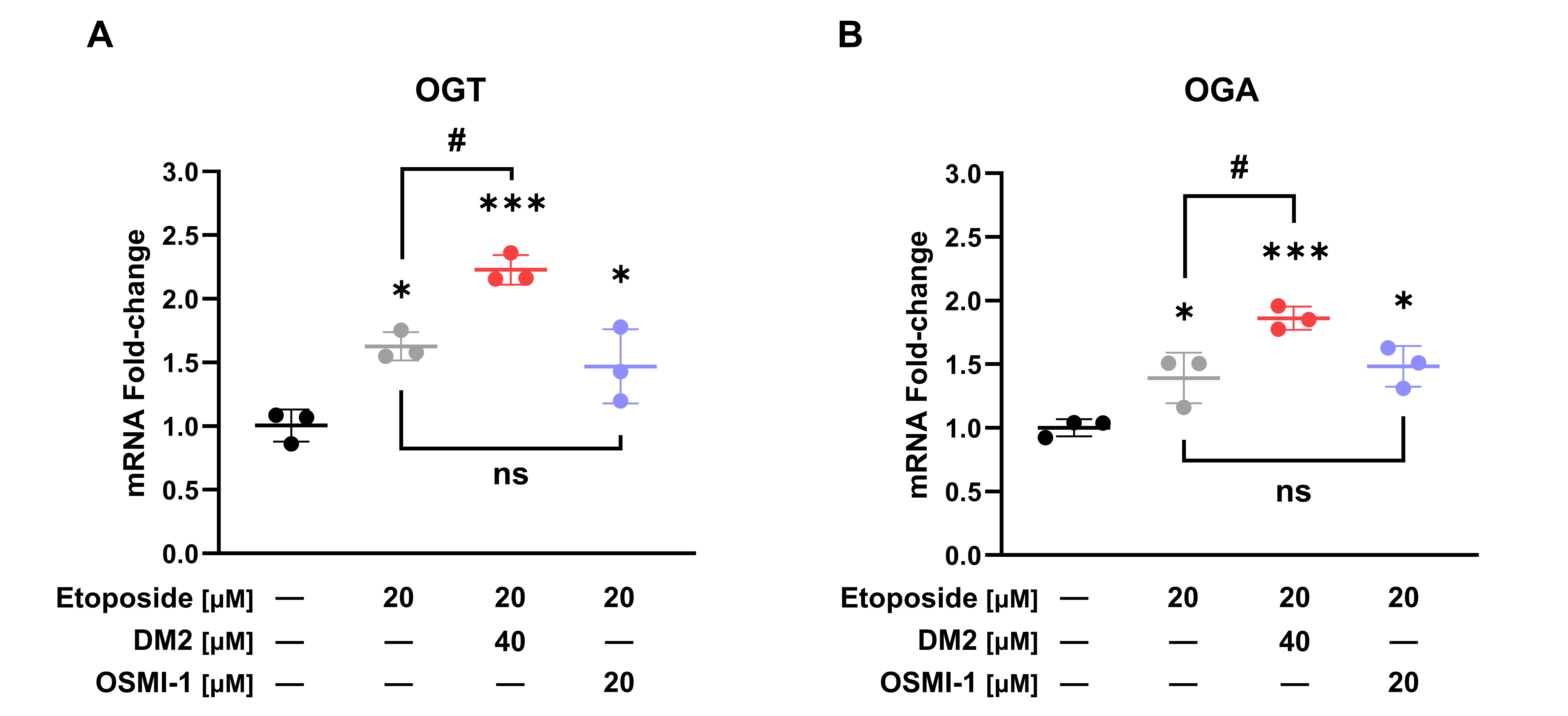


**Fig. S5. Effects of single and combined treatment with etoposide, DM2, and OSMI-1 on OGT and OGA mRNA expression in HepG2 cells.**

HepG2 cells were treated with the indicated compounds for 24 h. Total RNA was isolated, and RT-qPCR was performed using gene-specific primers. Relative expression levels were calculated using the ΔΔCt method and normalized to GAPDH. (A, B) Etoposide treatment increases the mRNA expression of both OGT and OGA, and this effect is further enhanced by co-treatment with DM2. However, cotreatment with OSMI-1 does not produce additional changes in OGT or OGA expression compared to etoposide treatment alone (*n* = 3). * *P* < 0.05 and *** *P* < 0.001 versus the control condition. # *P* < 0.05 for pairwise comparisons between matched conditions as indicated by connecting lines on the graph. Data represent means ± SD from three independent experiments. Statistical significance was determined with one-way repeated-measures ANOVA with post hoc Tukey test. ns, not significant.


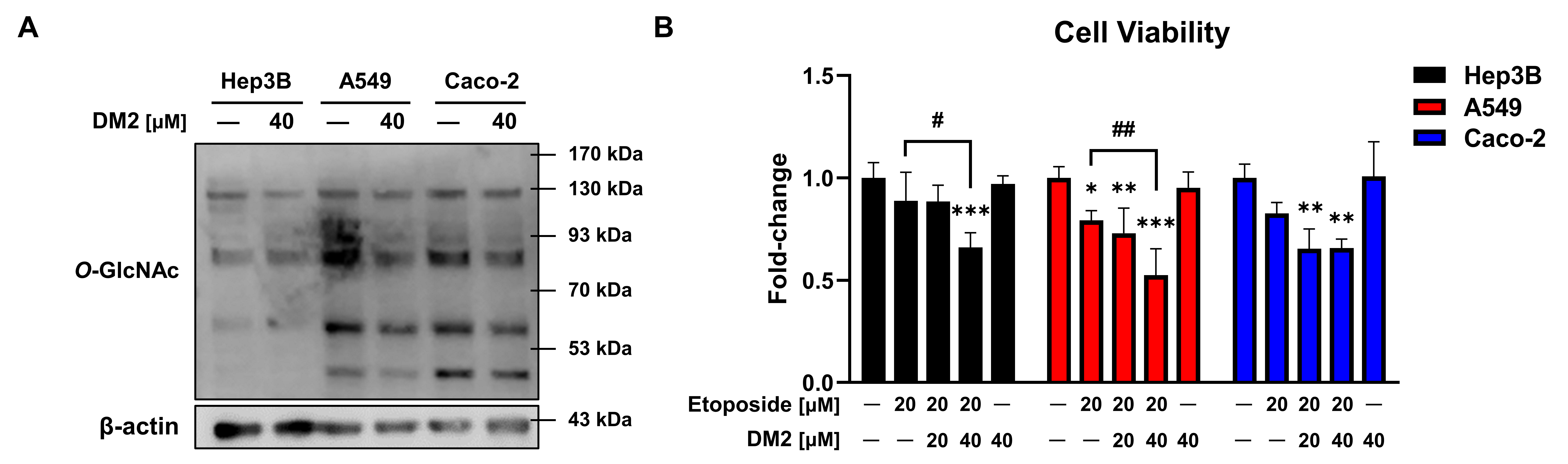


**Fig. S6. DM2 reduces *O*-GlcNAc levels and enhances etoposide-induced cytotoxicity and across various human cancer cell lines.**

(A) Analysis of Hep3B, A549, and Caco-2 cancer cell lines, showing reductions in *O*-GlcNAc levels consistent with those in HepG2 cells (*n* = 3). (B) Cell viability assay results indicating that DM2 significantly enhances the sensitivity of Hep3B, A549, and Caco-2 cells to etoposide (*n* = 4). * *P* < 0.05, ** *P* < 0.01, and *** *P* < 0.001 versus the control condition; # *P* < 0.05 and ## *P* < 0.01 versus the etoposide-only treatment condition. Data represent means ± SD from at least three independent experiments. Statistical significance was determined with one-way ANOVA with *post hoc* Tukey test.


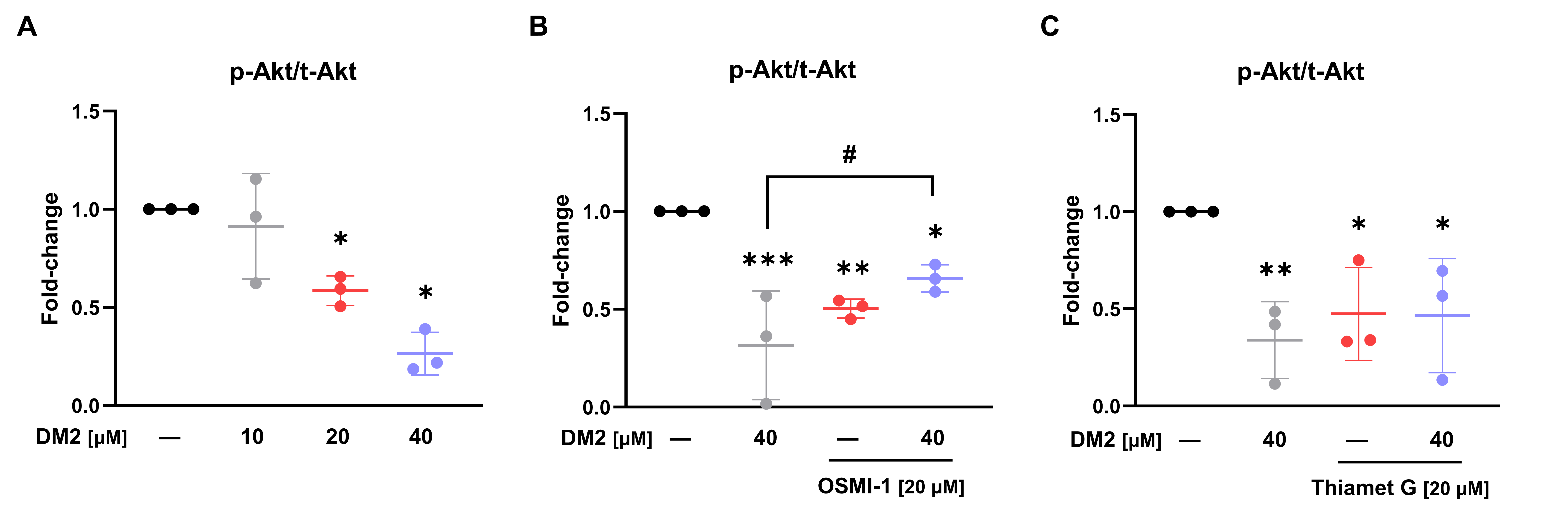


**Fig. S7. Quantitative evaluation of band intensities in the Fig. 7A–C western blot analysis.**

Densitometry (ImageJ) was performed on the bands shown in Fig. 7A–C. Band intensities were normalized to the corresponding total protein (t-Akt) and then expressed relative to the matched control within each experiment (control = 1.0 in every replicate). (A) p-Akt in Fig. 7A (*n* = 3). (B) p-Akt in Fig. 7B (*n* = 3). (C) p-Akt in Fig. 7C (*n* = 3). * *P* < 0.05, ** *P* < 0.01, and *** *P* < 0.001 versus the control condition; # *P* < 0.05 for pairwise comparisons between matched conditions as indicated by connecting lines on the graph. Data represent means ± SD from three independent experiments. (A) Statistical significance was determined with one-way repeated-measures ANOVA with post hoc Tukey test. (B and C) Statistical significance was determined with two-way repeated-measures ANOVA with post hoc Tukey test.


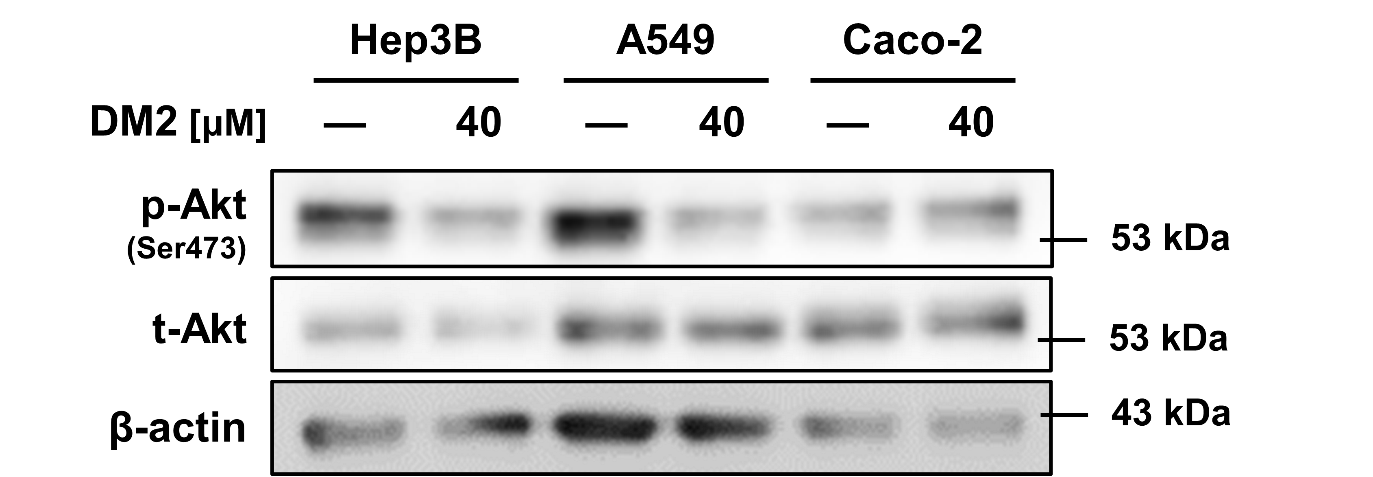


**Fig. S8. Differential modulation of Akt by DM2 across cancer cell lines.**

Similar inhibitory effects of DM2 on Akt observed in Hep3B and A549 cells but not in Caco-2 cells (*n* = 3).


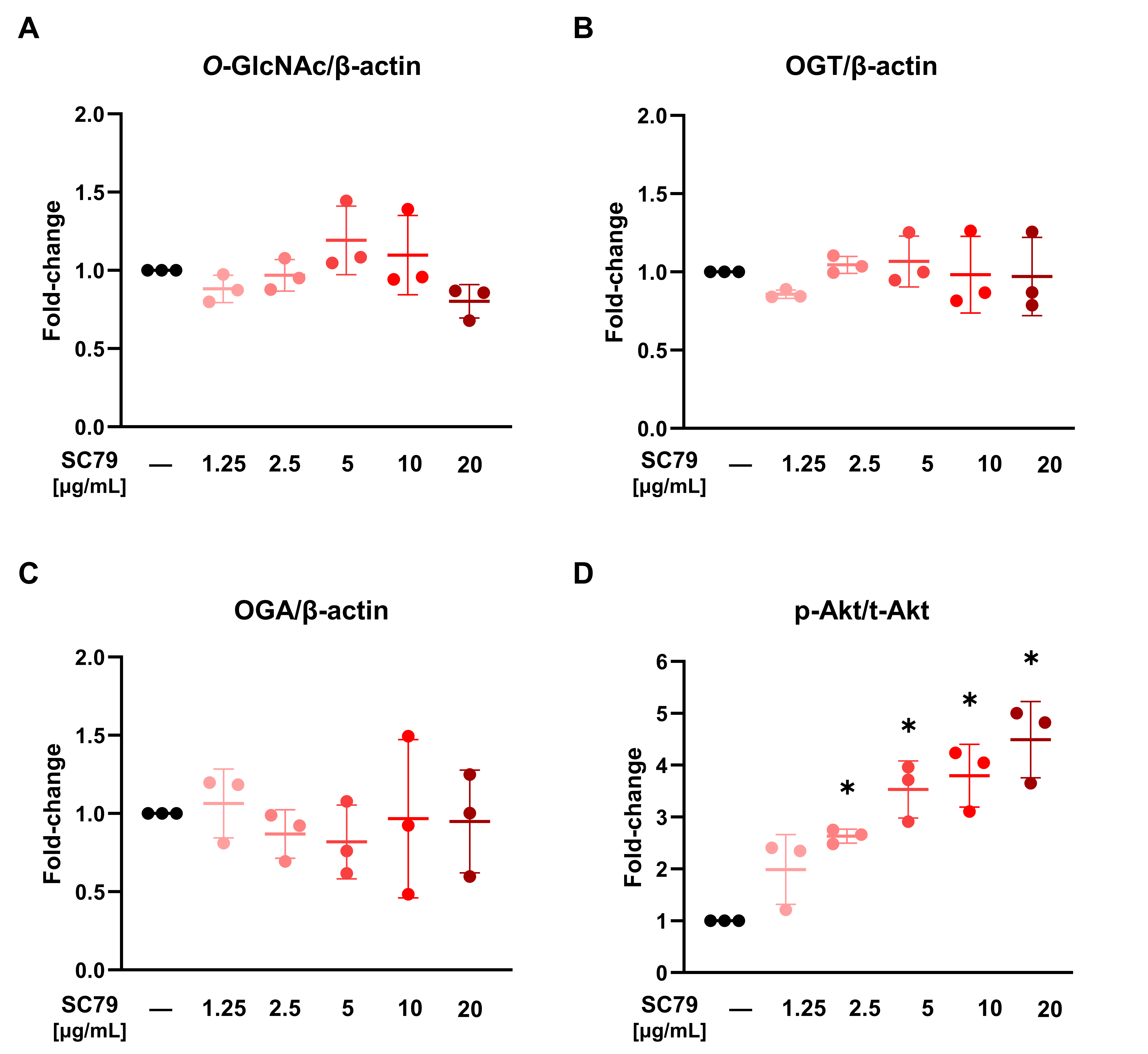


**Fig. S9. Quantitative evaluation of band intensities in the Fig. 8B western blot analysis.**

Densitometry (ImageJ) was performed on the bands shown in Fig. 7B. Band intensities were normalized to (A–C) the loading control (β-actin) or (D) corresponding total protein (t-Akt) and then expressed relative to the matched control within each experiment (control = 1.0 in every replicate). (A) *O*-GlcNAc (*n* = 3), (B) OGT (*n* = 3), (C) OGA (*n* = 3), (D) p-Akt (*n* = 3). * *P* < 0.05 versus the control condition. Data represent means ± SD from three independent experiments. Statistical significance was determined with one-way repeated-measures ANOVA with post hoc Tukey test.


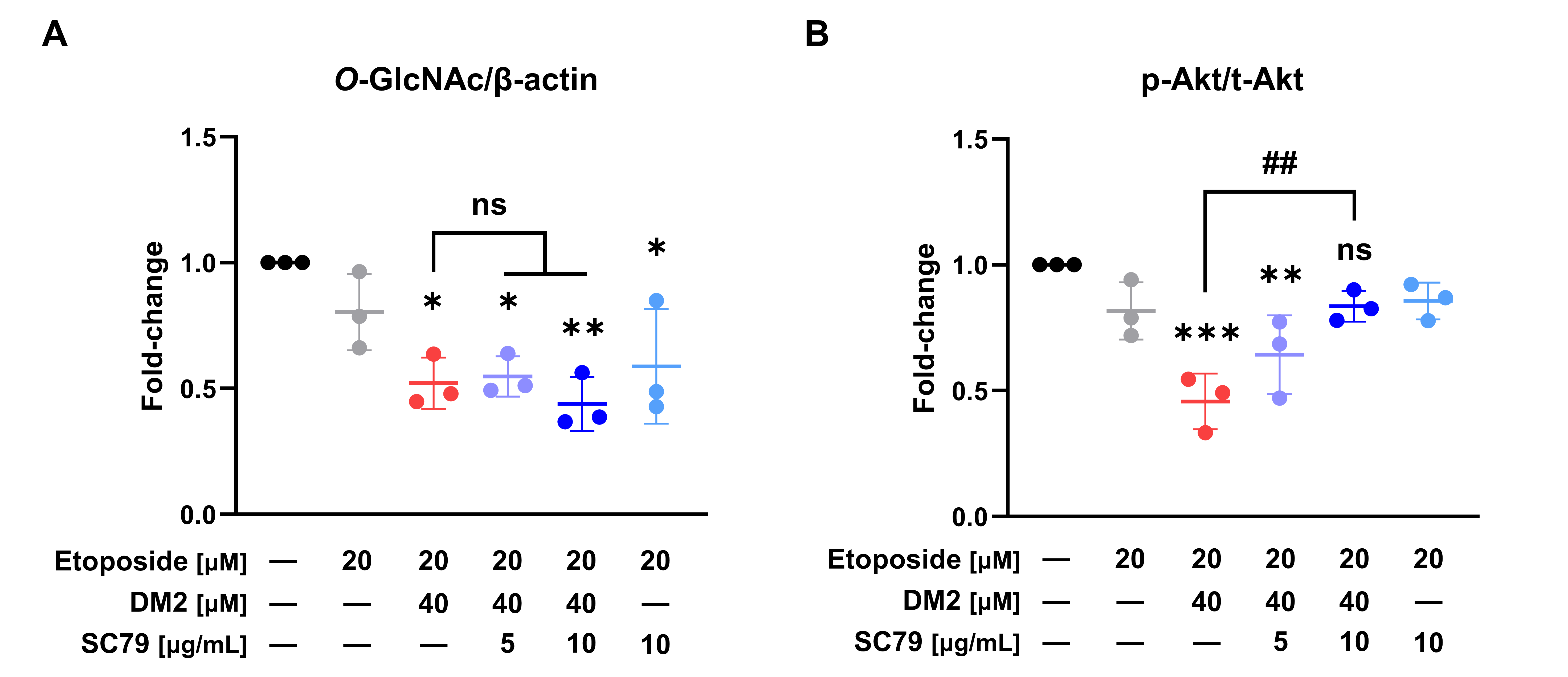


**Fig. S10. Quantitative evaluation of band intensities in the Fig. 8C western blot analysis.**

Densitometry (ImageJ) was performed on the bands shown in Fig. 7C. Band intensities were normalized to (A) the loading control (β-actin) or (B) corresponding total protein (t-Akt) and then expressed relative to the matched control within each experiment (control = 1.0 in every replicate). (A) *O*-GlcNAc (*n* = 3), (B) p-Akt (*n* = 3). * *P* < 0.05, ** *P* < 0.01, and *** *P* < 0.001 versus the control condition; ## *P* < 0.01 for pairwise comparisons between matched conditions as indicated by connecting lines on the graph. Data represent means ± SD from three independent experiments. Statistical significance was determined with one-way repeated-measures ANOVA with post hoc Tukey test. ns, not significant.


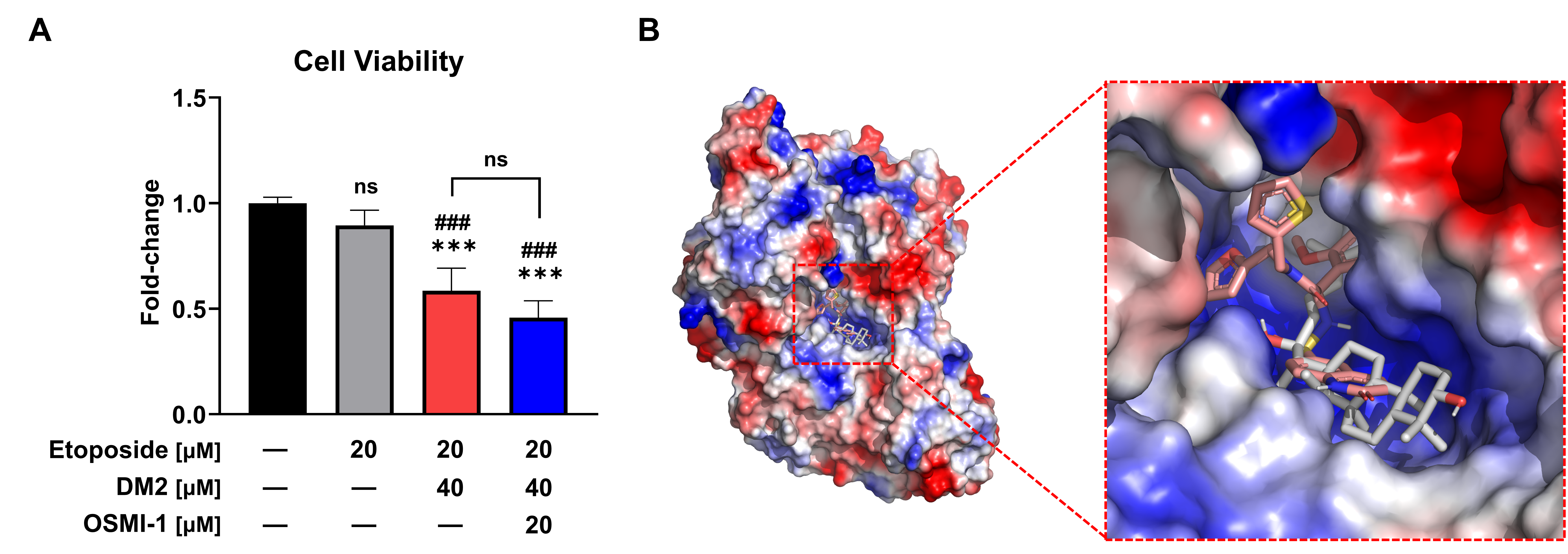


**Fig. S11. DM2 and OSMI-1 exhibit anticancer synergy through distinct binding orientations at the same OGT site.**

(A) Cell viability assay demonstrating a modest but not statistically significant reduction in viability upon combined treatment with etoposide, DM2, and OSMI-1 compared with etoposide and DM2 treatment. (*n* = 4). (B) The 3D electrostatic potential model generated using the Pharmaco-Net platform indicates that DM2 and OSMI-1 bind to the same binding site on OGT but occupy distinct positional orientations (pink molecule: OSMI-1; gray molecule: DM2). The red dashed box represents a magnified view of the molecular interactions between drugs and the active site of OGT *** *P* < 0.001 versus the control condition; ### *P* < 0.001 versus the etoposide-only treatment condition. Data represent means ± SD from at least three independent experiments. Statistical significance was determined with one-way ANOVA with *post hoc* Tukey test. ns, not significant.
