# Supplementary material for: Dammarenediol II enhances etoposide‐induced apoptosis by targeting O‐GlcNAc transferase and Akt/GSK3β/mTOR signaling in liver cancer
Source: Mol Oncol. 2025 Dec 30;20(6):1591–611. doi: 10.1002/1878-0261.70199 (PMC13238812; doi:10.1002/1878-0261.70199)
Supplement: Supplementary file 2 — Table S1. Primer sequences used for RT‐qPCR. [file MOL2-20-1591-s002.docx]

Supplementary Table

Table S1. Primer sequences used for RT-qPCR

|  | **Genes** | **5’ -> 3’** | **Sequence** |
| --- | --- | --- | --- |
| Human primers | OGT | Forward | CAGGAAGGCTATTGCTGAGAGG |
|  |  | Reverse | CGGAACTCACATATCCTACACGC |
|  | OGA | Forward | GCAAGAGTTTGGTGTGCCTCATC |
|  |  | Reverse | GTGCTGCAACTAAAGGAGTCCC |
|  | GAPDH | Forward | TGGACTCCACGACGTACTCA |
|  |  | Reverse | ACATGTTCCAATATGATTCC |
